# Carcinogenic and anticancer activities of microbiota-derived secondary bile acids

**DOI:** 10.3389/fonc.2025.1514872

**Published:** 2025-01-29

**Authors:** Mobina Kouhzad, Friedrich Götz, Tahereh Navidifar, Elahe Taki, Mahsa Ghamari, Roghayeh Mohammadzadeh, Maryam Seyedolmohadesin, Narjess Bostanghadiri

**Affiliations:** ^1^ Department of Genetics, Faculty of Science, Islamic Azad University North Tehran Branch, Tehran, Iran; ^2^ Department of Microbial Genetics, Interfaculty Institute of Microbiology and Infection Medicine Tübingen (IMIT), University of Tübingen, Tübingen, Germany; ^3^ Department of Basic Sciences, Shoushtar Faculty of Medical Sciences, Shoushtar, Iran; ^4^ Department of Microbiology, School of Medicine, Kermanshah University of Medical Science, Kermanshah, Iran; ^5^ Department of Pathobiology, School of Public Health, Tehran University of Medical Sciences, Tehran, Iran; ^6^ Department of Microbiology and Virology, School of Medicine, Mashhad University of Medical Sciences, Mashhad, Iran; ^7^ Department of Genetics, Faculty of Advanced Science and Technology, Tehran Medical Sciences, Azad University, Tehran, Iran; ^8^ Research Center for Clinical Virology, Tehran University of Medical Sciences, Tehran, Iran

**Keywords:** secondary bile acids, carcinogenic, anticancer, microbiota, bile acids

## Abstract

Secondary bile acids (SBAs), which are metabolites produced by gut microbiota, have been implicated in both carcinogenic and anticancer processes. This review explores the dual role of SBAs, focusing on their molecular mechanisms and biological effects. The carcinogenic activities of SBAs include DNA damage, promotion of oxidative stress, and modulation of signaling pathways that drive tumorigenesis. Conversely, some SBAs exhibit anticancer properties by inducing apoptosis, inhibiting cell proliferation, and modulating immune responses. The article also discusses the complex interplay between SBAs and the host’s genetic and environmental factors, highlighting potential therapeutic implications and the need for targeted strategies to mitigate risks while harnessing beneficial effects. A comprehensive understanding of the delicate equilibrium between the deleterious and salutary impacts of SBAs has the potential to facilitate the development of innovative cancer prevention and treatment methodologies.

## 1 Introduction

The microbiota, encompassing the microbiome or the collective genetic material of microorganisms, refers to the community of bacteria, viruses, fungi, and other microbes that inhabit a given ecosystem (1). The gut microbiota plays a central role in human health, facilitating the digestion and absorption of nutrients from food (2) and maintaining immune homeostasis by acting as a barrier against to pathogens. Imbalances in the microbiota, known as dysbiosis, are associated with conditions disease such as obesity, diabetes, inflammatory bowel disease, and neurological disorders. Furthermore, the gut microbiota contributes to overall gut health by producing beneficial compounds such as short-chain fatty acids with anti-inflammatory effects (3).

The gut microbiota plays a fundamental role in the well-being of its human host by assisting in the extraction of nutrients and producing metabolites that regulate host metabolic processes. An important category of these metabolites are BAs, which are synthesized in the liver from cholesterol and are essential for the digestion and absorption of dietary fats. Recent recognition of BAs as signaling molecules has highlighted their influence through receptors like the farnesoid X receptor (FXR) and the G protein-coupled membrane receptor 5 (TGR5) on the expression of genes involved in BA, lipid, and carbohydrate metabolism, as well as energy expenditure and inflammation. This regulatory effect is particularly pronounced in enterohepatic tissues but extends to peripheral organs (4).

Microbiome, actively participates in BA metabolism, transforming primary BAs into SBAs with distinct bioactive properties. This microbial modification not only influences lipid metabolism but also contributes to the broader regulation of physiological processes. Microbes in the lower gastrointestinal tract, particularly in the distal ileum, cecum, and colon, contribute to the chemical diversification of BAs through three main microbial pathways: deconjugation, dehydrogenation, and dehydroxylation reactions. Bile salt hydrolases (BSHs), which are prevalent in the gut microbiota, deconjugate host-derived primary bile acids, affecting the efficiency of fat emulsification, enterohepatic recirculation of BAs, and serum cholesterol levels. Microbial hydroxysteroid dehydrogenases (HSDH) further oxidize and epimerize specific hydroxyl groups on BAs, resulting in the formation of over 20 diverse metabolites. The physiological advantage of these microbial activities, especially BSHs, remains unclear. It has been suggested that microbially derived SBAs play a role in energy production, formation of less membrane-damaging bile acid pools, and modulation of enteric pathogen virulence. However, the impact on host health is complex, as some SBAs can be cytotoxic, contributing to oxidative stress, membrane damage, and colonic carcinogenesis, while others may exhibit anti-inflammatory and protective properties. Despite their pivotal role, much remains unknown about the specific gut microbes involved, the microbial biological functions, and the comprehensive impacts of SBAs on host health and disease. The bidirectional interaction between BAs and the gut microbiota suggests a nuanced relationship beyond digestion, potentially implicating microbiota-derived SBAs in the intricate network of carcinogenic and anticancer activities (5–7). There is extensive research on the carcinogenic and anticancer roles of SBAs. In a previous study, Zheng et al. (8) reviewed the role of deoxycholic acid (DCA) and lithocholic acid (LCA) as two main SBAs in inducing colon cancer. In addition, they reviewed the anti-cancer potential of short chain fatty acids derived from dietary fibers. Also, Yang and Qian (9) reviewed the direct and indirect role of DCA and LCA in cancer progression as strong signal molecules with a focus on the changes in cell cycle and signaling, as well as the inhibition of innate and specific immune system. In the present review, we described the role of SBAs in induction of cancer with a focus on DCA and LCA and anticancer role of some SBAs with an emphasis on ursodeoxycholic acid (UDCA) and tauroursodeoxycholic acid (TUDCA).

Understanding the carcinogenic and anticancer activities of microbiota-derived SBAs is crucial for unraveling the intricate interplay between the gut microbiota and human health. This exploration will provide insights into potential biomarkers for screening, prognosis, prediction and evaluation of treatment response. Moreover, the link between fat intake, BA production, and the impact on microbiota-derived SBAs underscores their broader implications for cancer risk and therapeutic considerations (10).

## 2 Microbiota and bile acid metabolism

### 2.1 Overview of primary bile acids

**Figure 1:**
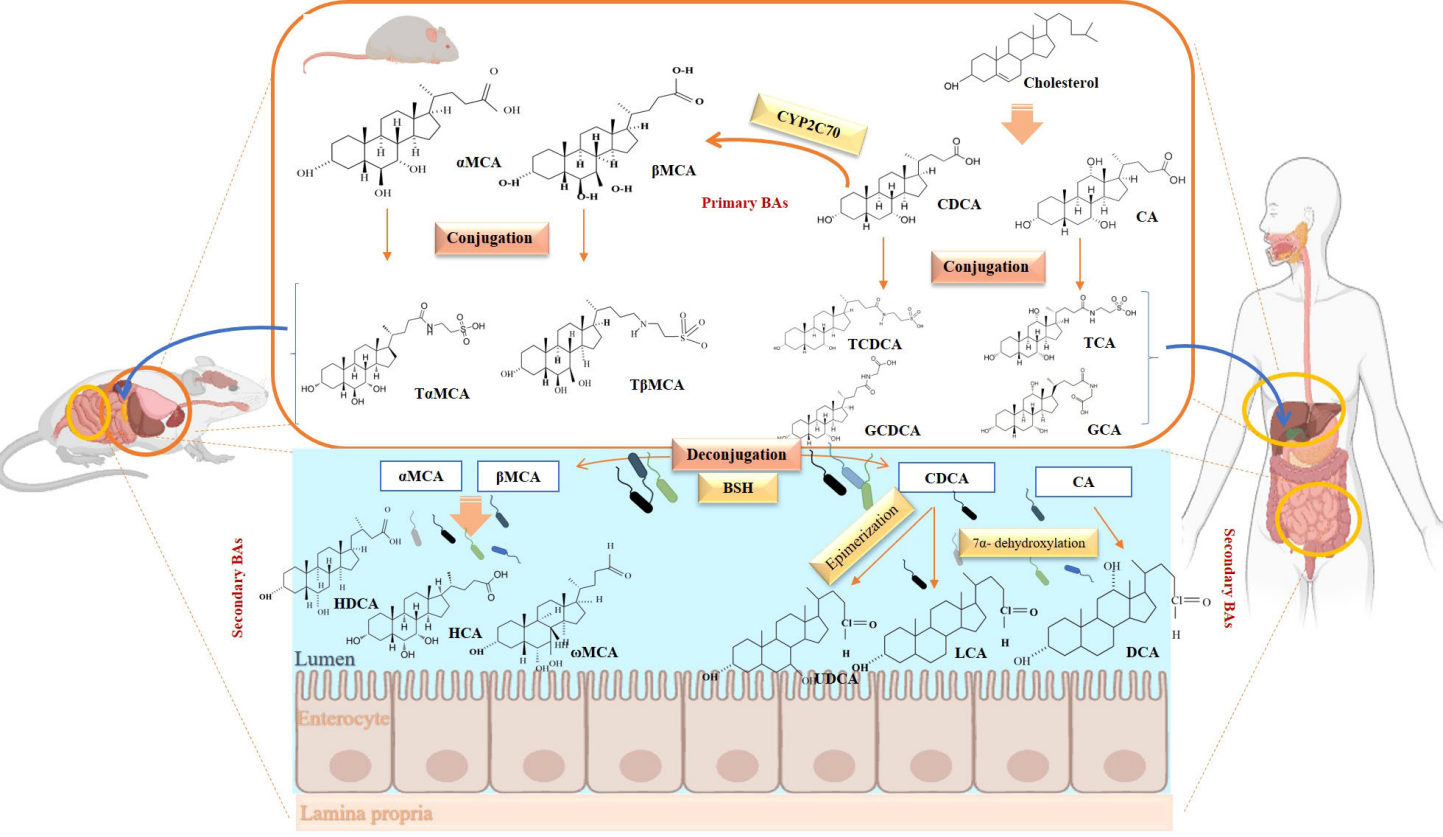
Overview of enterohepatic circulation of BAs in human and mouse. Cholesterol is converted to cholic acid (CA) and chenodeoxycholic acid (CDCA), which are the end products in the human liver, but in the mouse liver CDCA is further metabolized by CYP2C70 to muricholic acids (MCAs). In humans, bile acids are conjugated in the liver with glycine (G) or taurine (T) to form tauro-CA (TCA), tauro-CDCA (TCDCA), glyco-CA (GCA) and glycolyl-CDCA (GCDCA), whereas mice use almost exclusively taurine and produce tauro-α-muricholic acid (TαMCA) and tauro-β-muricholic acid (TβMCA). Primary bile acids are biotransformed by the gut microbiota. These reactions include deconjugation catalyzed by bile salt hydrolases (BSH), epimerization to change the orientation of the hydroxyl groups on the steroid nucleus of the bile acids, and 7-dehydroxylation. In humans, CA is converted to deoxycholic acid (DCA), while CDCA is converted to lithocholic acid (LCA) and ursodeoxycholic acid (UDCA), but in mice MCAs are converted to hyodeoxycholic acid (HDCA), hyocholic acid (HCA), omega-muricholic acid (ωMCA).

BAs are formed in in pericentral hepatocytes by a cascade of enzymatic processes using cholesterol as a substrate. Primary BAs, such as cholic acid (CA) and chenodeoxycholic acid (CDCA) in human, are synthesized in the liver by different pathways, the neutral and acidic pathways (4) (**Figure 1**). Initiation of the neutral pathway is facilitated by the enzyme Cholesterol 7α-hydroxylase (CYP7A1). This pathway then proceeds through two branches, namely the sterol 12α-hydroxylase (CYP8B1) and the sterol 27-hydroxylase (CYP27A1) and formed primary BAs, CA and CDCA, respectively. On the other hand, the acid pathway involves the conversion of cholesterol into 27-hydroxycholesterol by the action of CYP27A1 through a hydroxylation reaction. Subsequently, 27-hydroxycholesterol is transformed into CDCA by the enzyme oxysterol 7α hydroxylase (CYP7B1) (11, 12). CA and CDCA, are conjugated to either taurine or glycine through a covalent modification known as BAs to improve their solubility before being secreted into the lumen of the bile duct for concentration and storage in the gallbladder (13). Primary BAs are responsible for emulsifying dietary fats and activating pancreatic lipases in the small intestine (14). Primary BAs are subsequently reabsorbed by the enterocytes and passed through the portal circulation to the liver for reabsorption and reutilization. This cycle is referred to as the enterohepatic circulation of BAs (15). This cycle facilitates the reabsorption of 95% of BAs from the distal ileum, and only 5% are excreted into feces. The liver and intestine have a precise system for regulating the level of the bile acid pool and preventing the harmful accumulation of BAs (16). Humans and mice have different bile acid compositions, which are synthesized by different mechanisms. These differences between the human and mouse species are summarized in **Figure 1** and **Table 1**.

**Table 1:** Diversity of known bile acids.

Type	Bile acid	Abbreviation	Structure	Source	R1	R2	R3	R4	R5
**Primary**	Cholic acid	CA	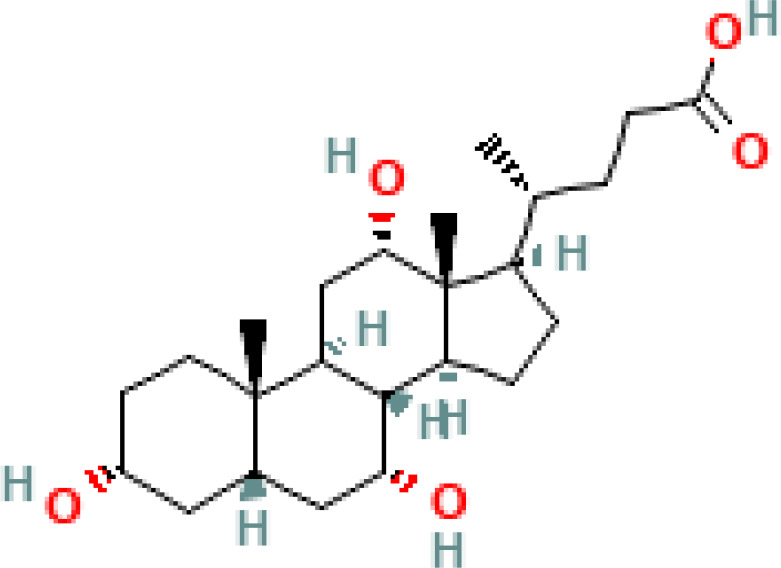	Human	H	H	OH	H	OH
	Chenodeoxycholic acid	CDCA	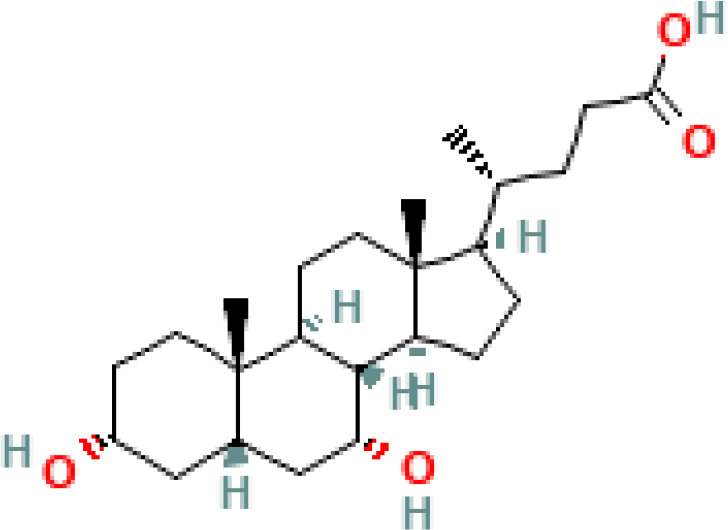	Human	H	H	OH	H	H
	α-muricholic acid	αMCA	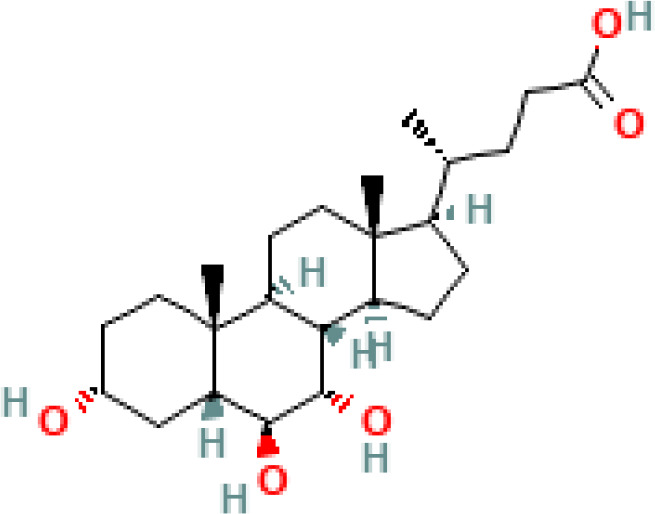	Mice	H	OH	OH	H	H
	β-muricholic acid	βMCA	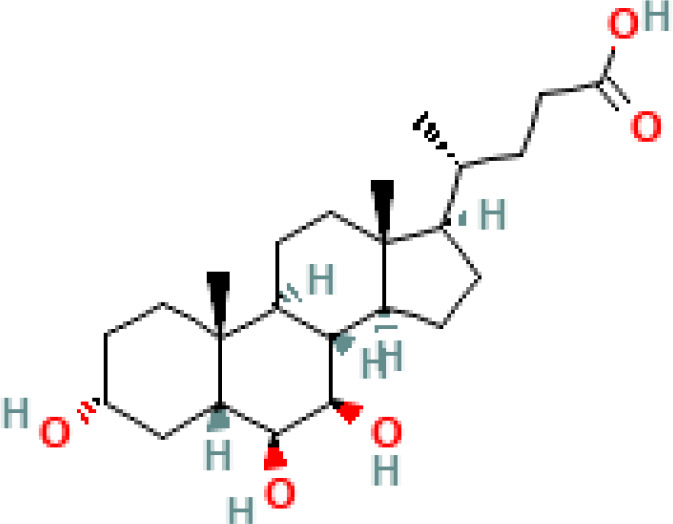	Mice	H	OH	H	OH	H
	Ursocholic acid	UCA	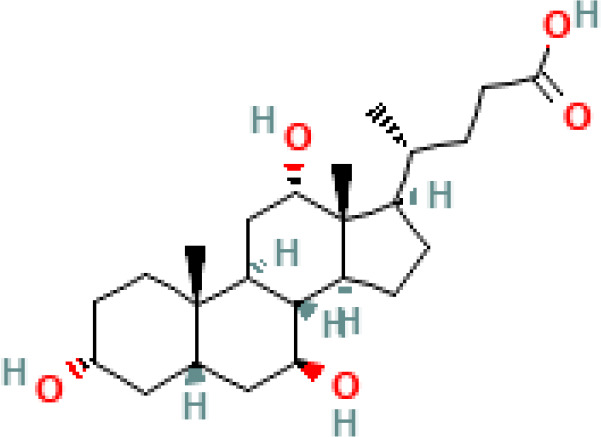	Human	H	H	H	OH	H
	7-Epicholic acid	7-ECA	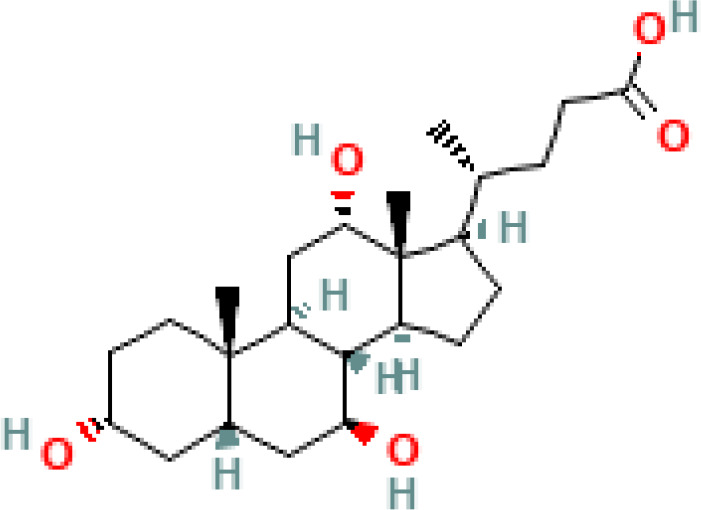	Human	H	H	OH	H	H
	Glycocholic acid	GlyCA	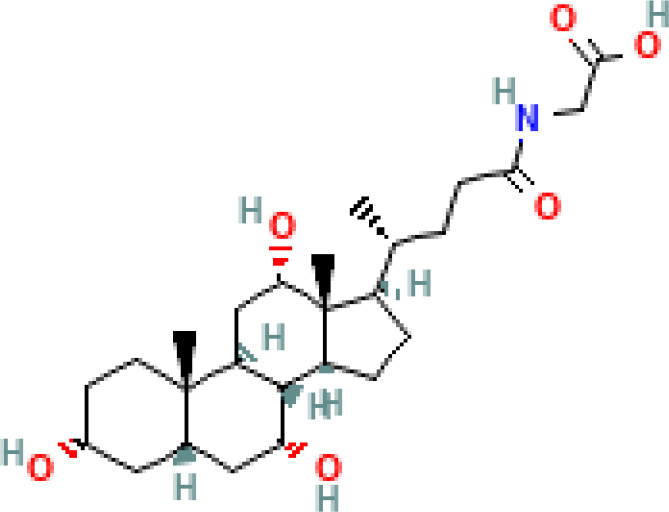	Human	α	H	α	α	Glycine
	Taurocholic acid	TaurCA	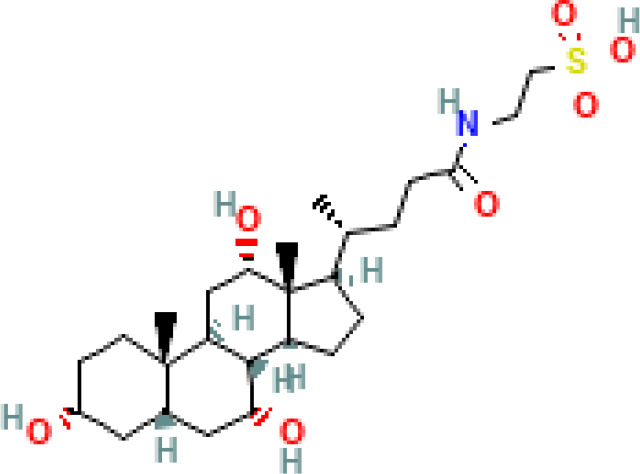	Human	α	H	α	α	Taurine
	Glycochenodeoxycholic acid	GlyCDCA	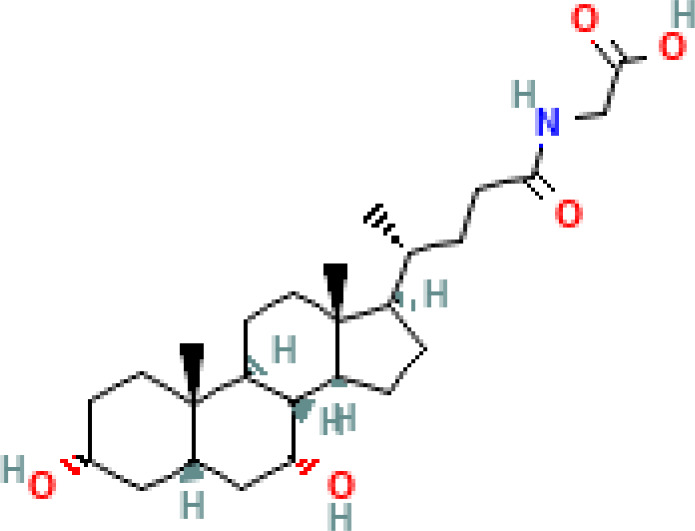	Human	α	H	α	H	Glycine
**Secondary**	Deoxycholic acid	DCA	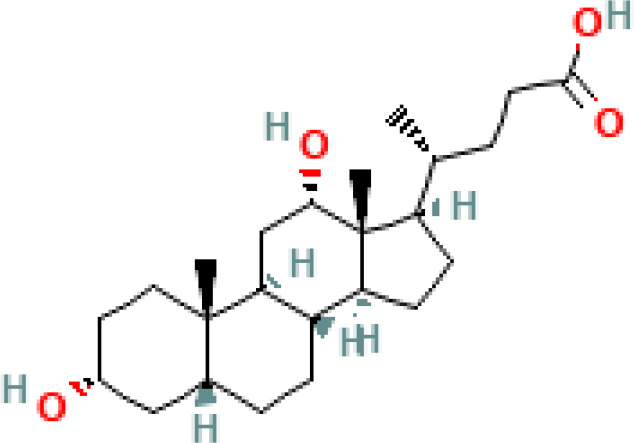	Human	H	H	H	H	OH
	Lithocholic acid	LCA	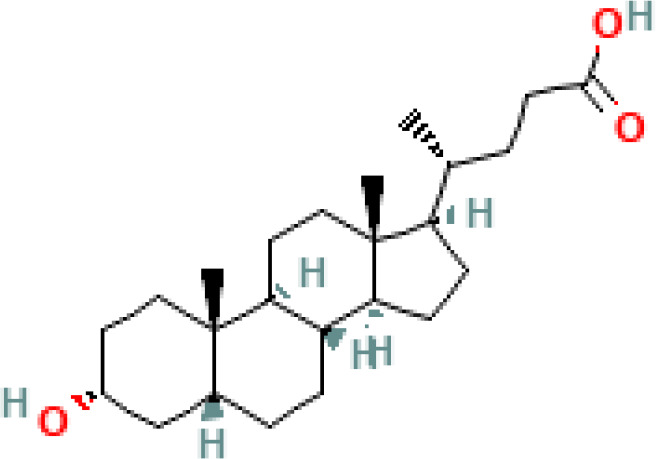	Human	H	H	H	H	H
	Ursodeoxycholic acid	UDCA	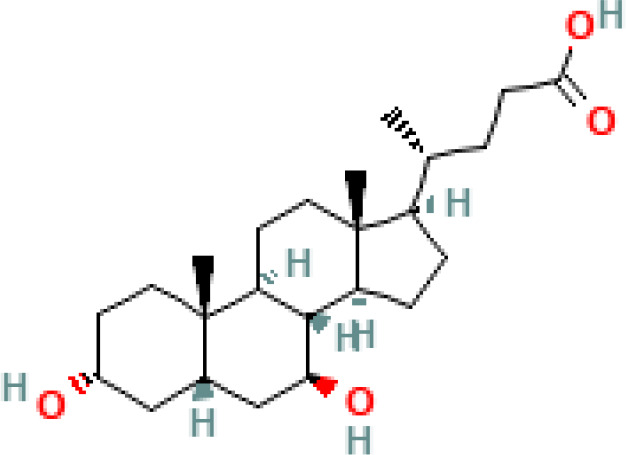	Human	H	H	H	OH	H
	Hyodeoxycholic acid	HDCA	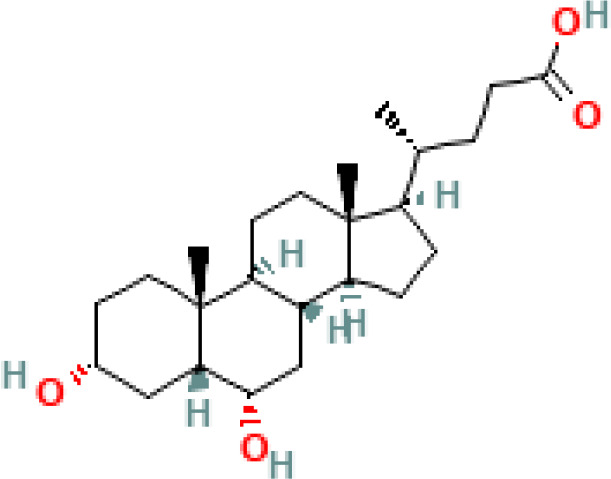	Mice	OH	H	H	H	H
	Hyocholic acid	HCA	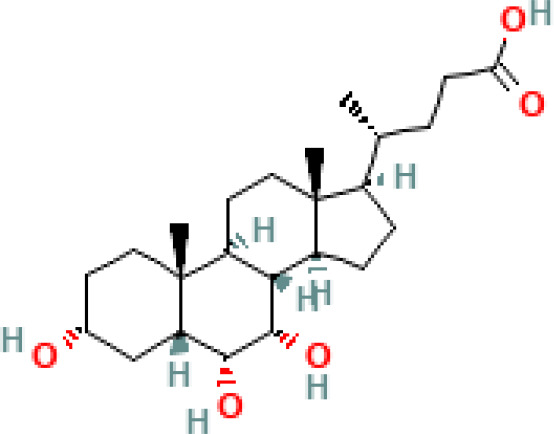	Pig	OH	H	OH	H	H
	ω- muricholic acid	ωMCA	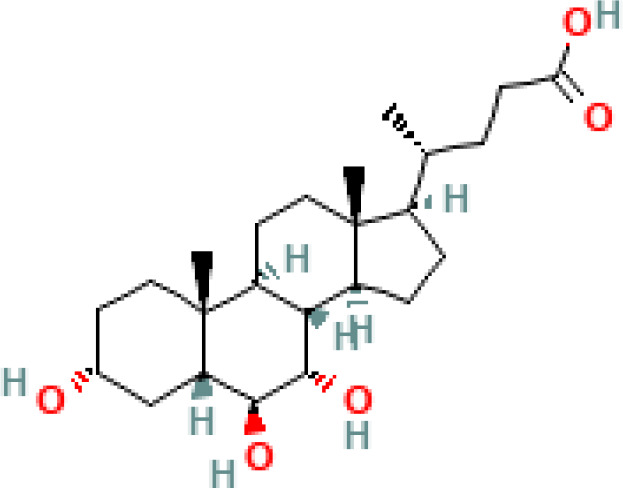	Mice	OH	H	H	OH	H
	Allolithocholic acid	alloLCA	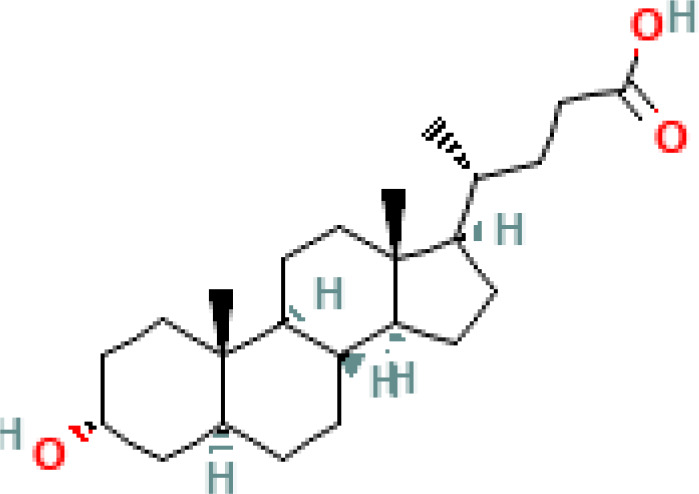	human	α	H	H	H	OH
	Allodeoxycholic acid (alloursodeoxycholic)	alloDCA	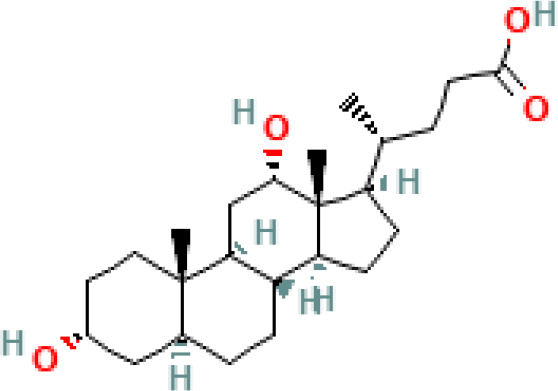	human	α	H	H	α	OH
	7-epicholic acid	7-ECA	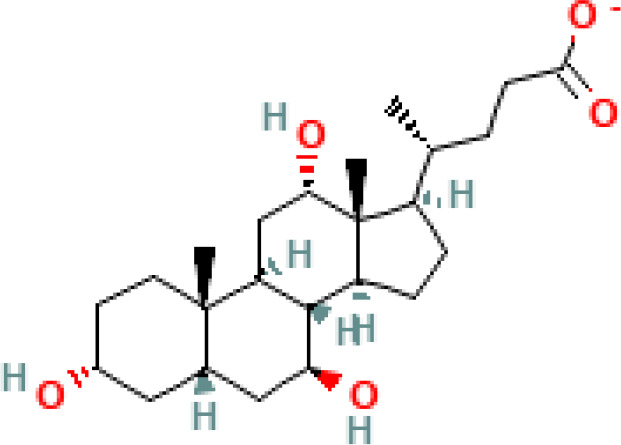	Cows/pigs	α	H	β	α	OH
	Isochlocic acid (ursocholic acid)	iCA	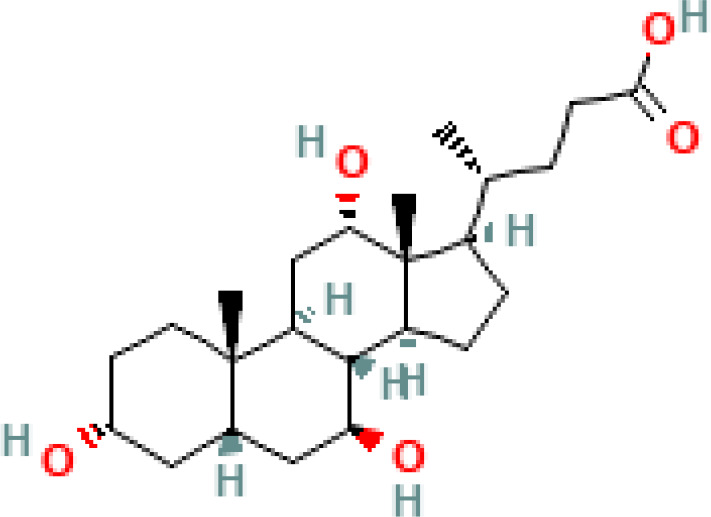	Poultry/bovine	β	H	α	α	OH
	Isochenodeoxycholic acid	iCDCA	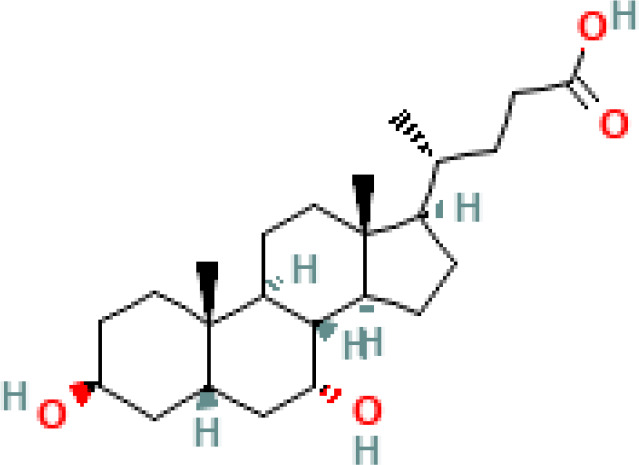	human	β	H	α	H	OH
	isodeoxycholic acid	iDCA	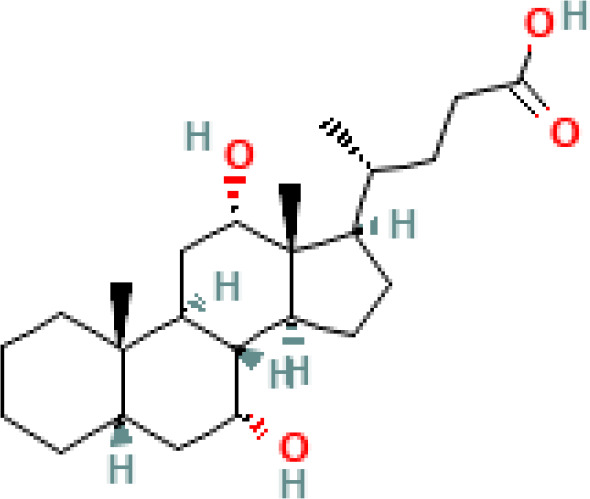	human	β	H	H	α	OH
	Isolithocholic acid	iLCA	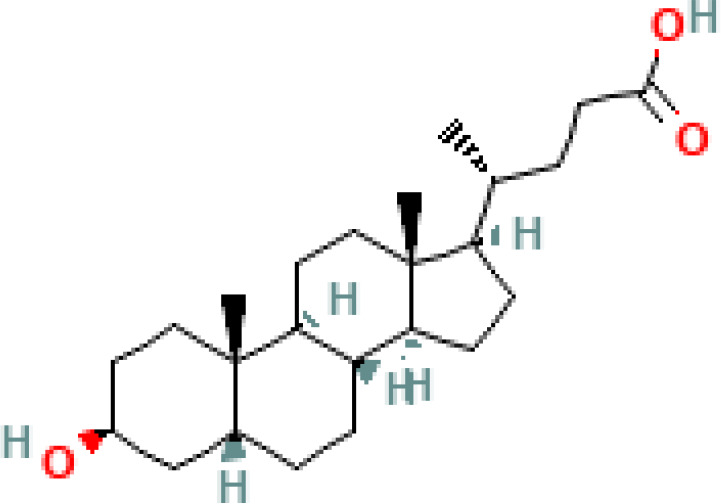	human	β	H	H	H	OH
	12-oxoisochenodeoxycholic acid	12-oxoCDCA	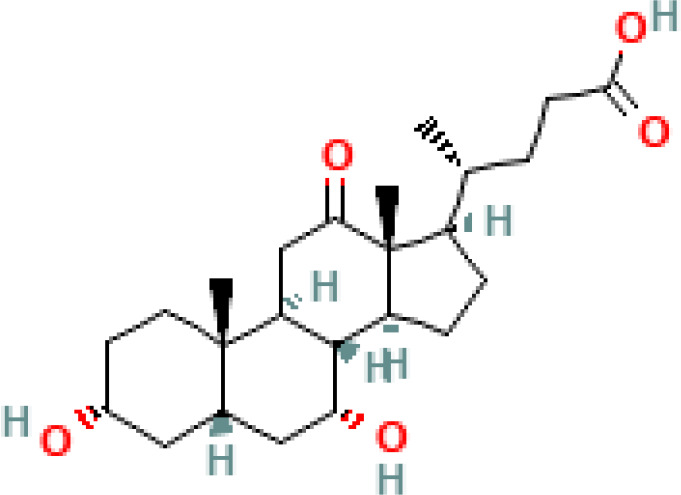	human	α	H	α	oxo	OH
	7-oxodeoxycholic acid	7-oxoDCA	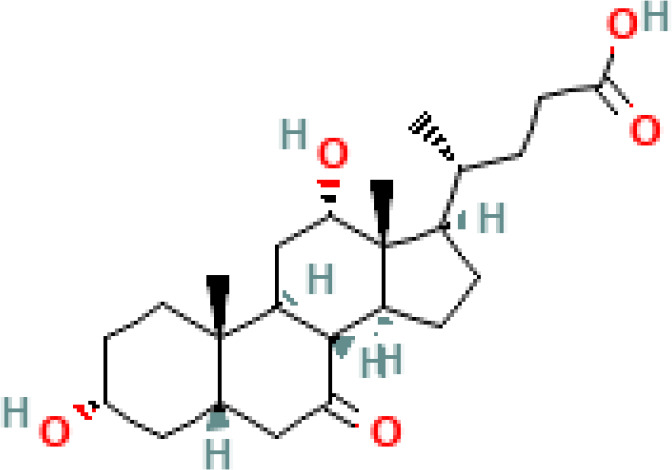	human	α	H	oxo	α	OH
	3-oxocholic acid	3-oxoCA	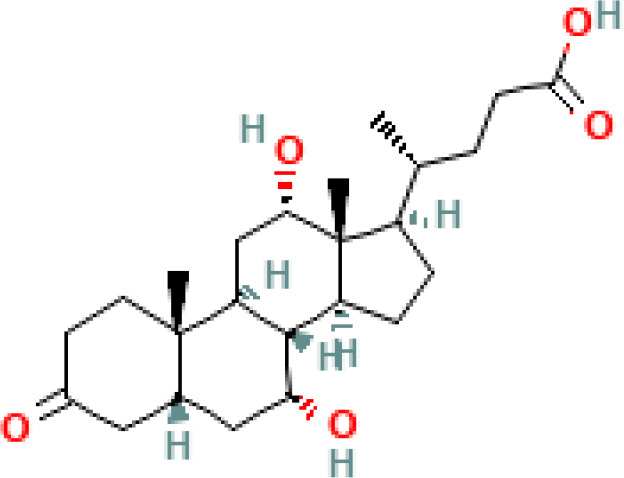	human	oxo	oxo	α	α	OH
	7-oxolithocholic acid	7-oxoLCA	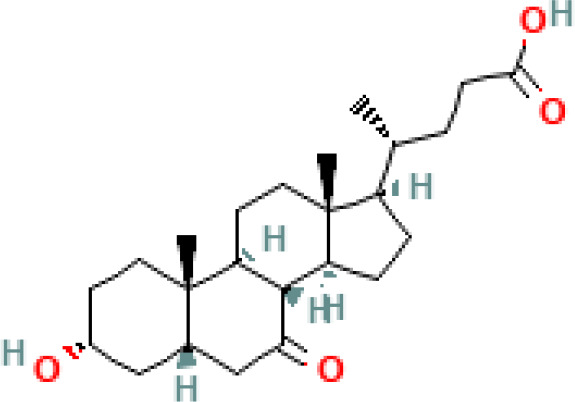	human	α	H	oxo	H	OH
	3- oxochenodeoxycholic acid	3-oxoCDCA	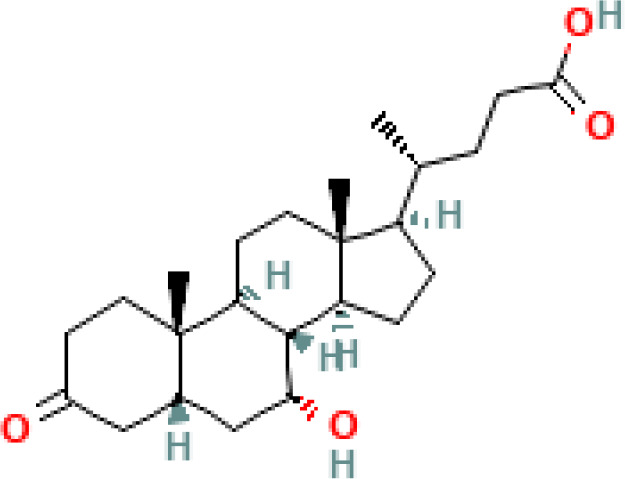	human	oxo	H	α	H	OH
	Phenvlalanocholic acid	PheCA	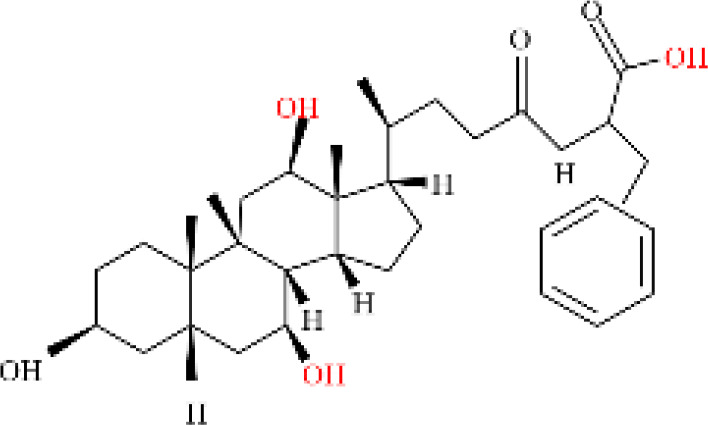	human	α	H	α	α	Phenylalanine
	Tyrosocholic acid	TyrCA	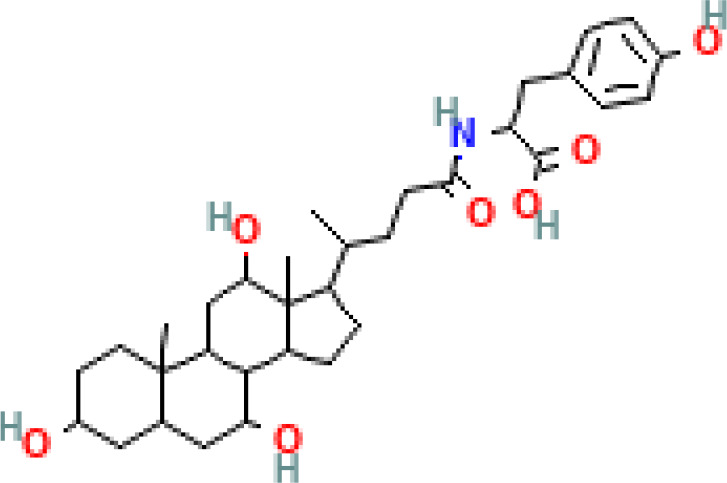	human	α	H	α	α	Tyrosine
	Leucholic acid	LeuCA	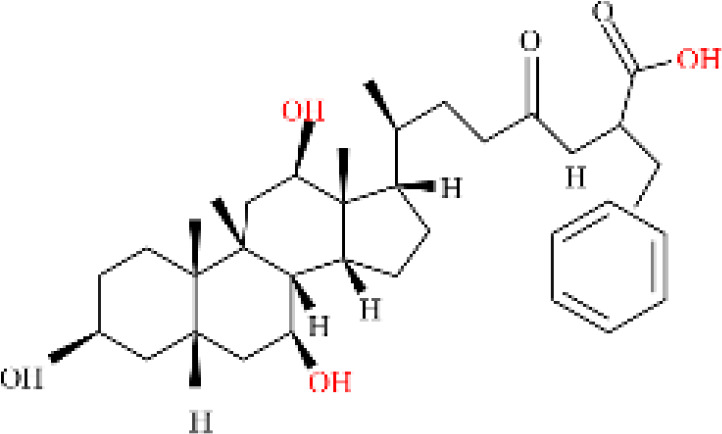	human	α	H	α	α	Leucine

### 2.2 Synthesis of secondary BAs

In the terminal ileum, cecum, and upper colon conjugated primary BAs are catalyzed by intestinal bacteria to SBAs through various mechanisms like deconjugation, dehydroxylation, oxidation, isopropylation, desulfurization, and esterification (9) (**Figure 1**). Bacterial enzymes responsible for SBAs production are clustered in the BA-inducible (bai) operon (17).

#### 2.2.1 Microbial deconjugation

The process of deconjugation of primary BAs is commonly referred to as the “initial reaction” that leads to further modification (18). Various theories have been proposed to elucidate the significance of deconjugation. As a result, the microbiota may have evolved the deconjugation process as a means of controlling BA production (19). BSHs secreted by gut bacteria, classified as EC 3.5.1.24, are involved in the deconjugation of conjugated primary BAs and reconverts them to free primary BAs CA and CDCA (20). The ability to deconjugate BAs is a common characteristic of both aerobic and anaerobic bacteria found in the small and large intestine (21). The Firmicutes and Bacteroidetes phyla are the most common bacteria with BSHs activity (22). Gram-positive bacteria include *Clostridium, Lactobacillus, Bifidobacterium, Enterococcus*, and *Listeria* establish the majority of the BSHs bacteria category (23) while the *Bacteroides* genus is the only group of Gram-negative bacteria with BSHs activity (24, 25) *Methanobrevibacter smithii* and *Methanosphera stadtmanae* from class Archaea also possess BSH enzymes that can degrade both taurine and glycine-bound compounds (21). According to Jones and colleagues’ metagenomic research, BSH-encoding genes are ubiquitous in the gut microbiome of both bacterial and archaeal species. They also found that 26.03% of the identified strains of human gut bacteria possess BSHs activity (18).

#### 2.2.2 Microbial 7-alpha-dehydroxylation

The processes of BA deconjugation and dehydroxylation are distinct, but may be linked by regulatory pathways. Following deconjugation by the action of BSH and the production of free primary BAs, a multistep process known as 7-α-dehydroxylation leads to the production of SBAs (DCA, LCA and UDCA) (26). In the aforementioned procedure, CA is transformed into DCA, while CDCA is transformed into LCA and UDCA. LCA, DCA are the most abundant SBAs, and UDCA is found at lower levels in humans (27, 28). Certain bacteria possess the ability to remove hydroxyl groups from unattached BAs, although only a limited number exhibit 7α-hydroxysteroid dehydrogenase (7α-HSDH) activity (29). These bacteria are predominantly found in the phylum Firmicutes, specifically *Clostridium* and *Eubacterium*. Furthermore, both *Ruminococcus* and *Trichospiraceae* exhibit 7α-HSDH activity, and there is a direct relationship between the abundance of *Ruminococcus* and the level of DCA (30). A recent study showed that *Desulfovibrionales* also have 7α-HSDH activity and that mice carrying *Desulfovibrionale* produced more SBAs (31).

#### 2.2.3 Oxidation and epimerization

Other reactions of the production of SBAs are oxidation and epimerization, which may be associated with intestinal Firmicutes (*Clostridium, Eubacterium*, and *Ruminococcus*), *Bacteroides*, and *Escherichia* (32).

The epimerization process occurred by the gut microbes plays an important role in expanding the chemical diversity of SBAs. Two distinct steps are involved in this process: a position-specific hydroxysteroid dehydrogenase oxidizes the hydroxyl group which can yield derivatives such as ursocholic acid, 12-epicholic acid, or isocholic acid from CA, while epimerization of CDCA can lead to the formation of either UDCA or isochenodeoxycholic acid (33). The diversity within unconjugated BAs can be attributed to the occurrence of oxidation and subsequent epimerization at all three CA hydroxyl positions and both CDCA hydroxyl positions. The 7α-epimerization of UDCA is facilitated by *Clostridium baratii* and other unknown isolates. Studies have shown that *C. baratii* can convert CDCA to UDCA, but lacks the ability to convert glyco- and tauro-BAs, instead degrading taurCDCA prior to epimerization (34). The production of UDCA is highly dependent on the independent epimerization of CDCA without the involvement of conjugation. The conversion of 7-oxo-LCA to UDCA is facilitated by the actions of *Ruminococcus gnavus*, *Clostridium absonum*, *Stenotrophomonas maltophilia*, and *Collinsella aerofaciens* using either NADH or NADPH (35–37). While the primary function of the gut microbiota is to reduce BAs oxidized at a single position, it also has the potential to reduce BAs oxidized at two or three positions. Other members of the *Coriobacteriaceae* family, including *C. aerofaciens, E. lenta*, and *Lancefieldella parvula*, have also shown comparable patterns of non-target hydroxyl oxidation. Some members did not oxidize DCA at both C3 and C12, while all strains capable of modifying DCA were found to oxidize at both positions (38). This suggests that oxidation is a potential method for microbes to detoxify BAs. As amphipathicity decreases, oxidized BAs gradually lose their ability to act as detergents, protecting DNA and membranes from damage (19).

## 3 Types of secondary bile acids produced by microbiota

More than 50 types of SBAs, produced by microbes, can be found in human feces. However, the most common ones are DCA and LCA. There are other SBAs which are derivates LCA and DCA, including, Glycodeoxycholic acid (GDCA), Glycolithocholic acid (GLCA), Glycoursodeoxycholic acid (GUDCA), Taurodeoxycholic acid (TDCA), Taurolithocholic acid (TLCA), TUDCA, Hyodeoxycholic acid (HDCA), and UDCA. Among them, DCA and GDCA have the highest levels of serum SBAs (7). In human feces, although DCA and LCA are predominate (39). Unabsorbed SBAs are excreted from the body in the feces (40). DCA is capable of being absorbed again in the colon, returned to the liver, and reintroduced into the bile acid pool within the body. LCA is less absorbed in the colon and is mostly excreted in the feces rather than returning to the bile acid pool. The potential diversity of the human conjugated BA pool can be enhanced 5 times by restricting the bile acid backbone to only those conjugated by the host (CA and CDCA) and limiting the conjugated amino acids to those naturally found in humans.

## 4 Factors influencing the composition and levels of secondary bile acids

Fecal BA levels can be influenced by a number of dietary factors, including total energy intake, type and amount of dietary fat, and dietary fiber. In addition to diet, physical activity may play a role in modifying BA concentrations, providing insight into why exercise is associated with a lower risk of colon cancer (41, 42). Results from a basic model indicated that greater levels of physical activity are associated with lower concentrations of fecal BAs, and this association becomes more robust as the duration of physical activity increases (41).

High-fat diets lead to increased primary BA release, resulting in higher concentrations of SBAs in the colon compared to low-fat or normal-fat diets (43). In another study, a diet high in milk-derived saturated fat was associated with an increase in taurine-conjugated BAs, which promote the growth of potentially harmful bacteria in the gut microbiome (44). It is evident that colonic BAs have a significant impact on the composition of the gut microbiome (8). There is a dynamic interplay between host BAs and the gut microbial population. For example, when rats were given CA at mM concentrations (equivalent to a high-fat diet), their microbiota composition at the phylum level was significantly altered, with an increase in *Firmicutes* and a decrease in *Bacteroidetes*. This highlights the significant influence of colonic BAs on the composition of the gut microbiome. Several studies conducted in human populations, including those focused on colorectal cancer (CRC) patients, have shown that a high-fat diet leads to increased levels of SBAs in feces, primarily DCA and LCA (45, 46). In addition, the potential of BAs to act as tumor promoters has been investigated in a mouse model by a variety of experimental methods, including pretreatment, co-administration, or post-treatment with carcinogens (47). A high-fat diet is linked to numerous digestive diseases and tumors. It may also accelerate the development of cancer through inflammation and metabolic changes. A high-fat diet can alter the composition of BAs in mice, particularly taurocholic acid (TCA) and TUDCA, which may lead to an increased incidence of Barrett’s esophagus and esophageal cancer in these animals (48). Research has shown that a high-fat diet can lead to a marked increase in the retention of hydrophobic BAs in the liver, which is closely linked to changes in the gut microbiota. In addition, the production and transport of bile acids in the liver are disrupted, resulting in the release of several inflammatory cytokines and significant bile acid accumulation, which may contribute to the development of liver cancer (49). One study showed that healthy rural Africans, who have a very low incidence of CRC at less than 5 cases per 100,000 and eat a low-fat, high-fiber diet, have lower levels of BAs and 7α-dehydroxylating bacteria in their feces than healthy African Americans. The latter group, which consumes a high-fat, low-fiber diet, has the highest rate of CRC in the contiguous United States, with an incidence of 65 cases per 100,000 (50). Importantly, the tumor-promoting effects of the altered gut microbiota influenced by a high-fat diet or DCA, along with the host’s genetic predisposition, were transferable when the fecal microbiota was transplanted into K-ras or ApcMin/+ mice fed a standard diet. This suggests that dietary fats and BAs have a lasting effect on the composition and function of the gut microbiota in relation to colon tumor development (51, 52). The increase in colonic DCA and LCA levels leads to apoptosis primarily through the stimulation of intrinsic apoptotic pathways, including mitochondrial oxidative stress, reactive oxygen species (ROS), cytochrome C, and cytosolic caspases (8). The increased incidence of fecal BA and CRC in European and American populations can be attributed to their high-fat, high-protein diets, while Asian and African populations, whose diets are lower in fat and protein and higher in cellulose, have lower rates of these conditions (53). In all these groups, the concentration of fecal BA and the prevalence of CRC are elevated in meat eaters compared to vegetarians. Several studies have shown that the concentrations of DCA, LCA, UDCA and other indicators in the feces of CRC patients were higher than those of the normal control group, while the levels of primary BAs (CA, CDCA) were not different from those of the normal control group (53).

## 5 Carcinogenic activities of microbiota-derived secondary bile acids

**Figure 2:**
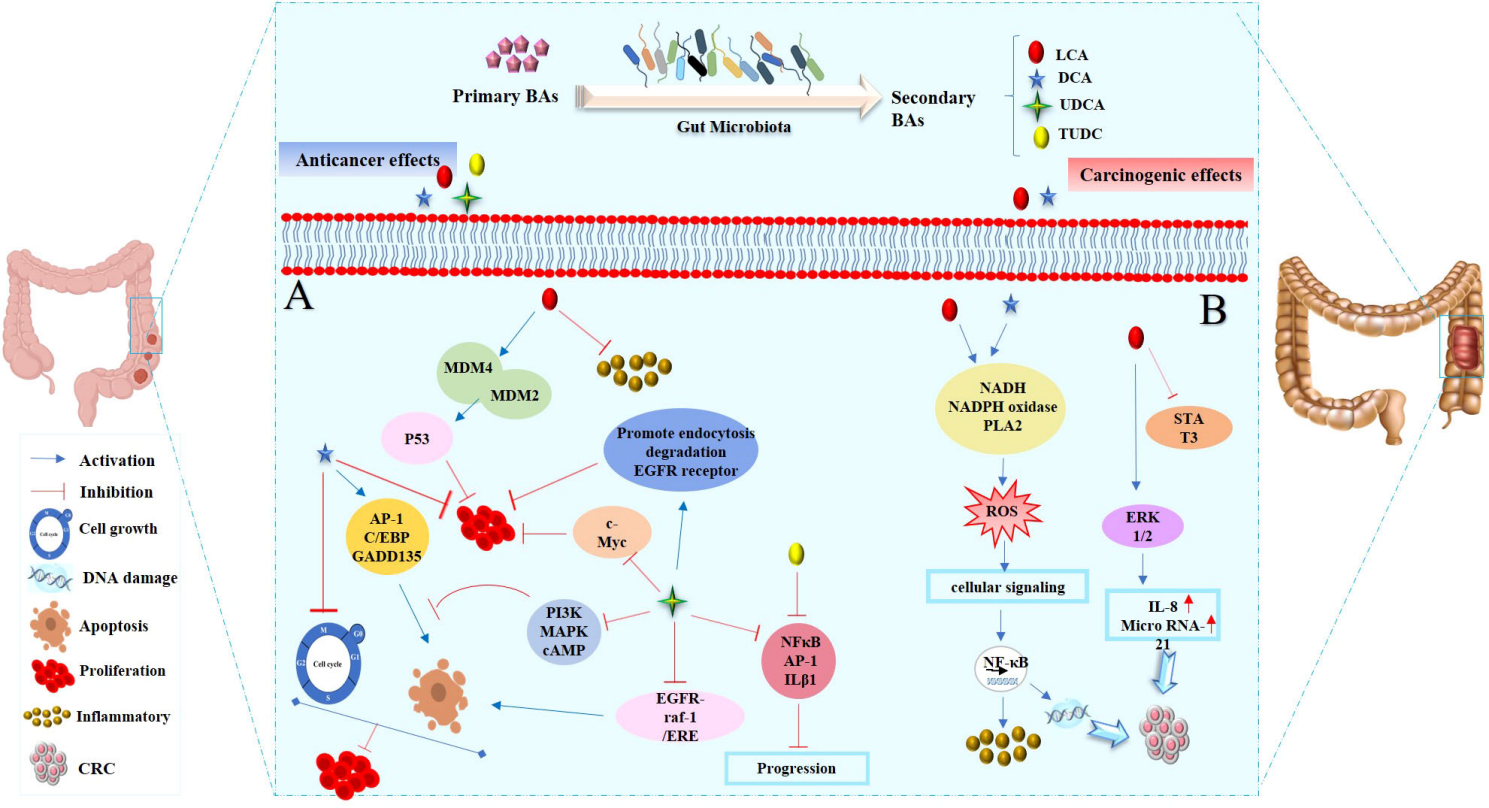
The dual roles of SBAs in colon cells: anticancer and cancer-promoting effects. **(A)** Anticancer effects of SBAs in colorectal cancer (CRC): LCA inhibits inflammatory signals and activates p53, which binds to MDM4 and MDM2, thereby preventing cell proliferation. DCA induces the expression of apoptosis-related genes (GADD153, AP-1, and C/EBP), key regulators of the cell cycle and apoptosis. UDCA suppresses c-Myc expression, promotes endocytosis and degradation of EGFR, and blocks PI3K, MAPK, or cAMP pathways to induce apoptosis. Partially inhibits DCA-induced apoptosis by disrupting the EGFR/Raf-1/ERK signaling pathway. Both UDCA and TUDCA inhibit interleukin β1, NF-κB, and AP-1 activation. **(B)** Cancer-promoting effects of SBAs in CRC development: DCA and LCA are major pathogenic factors that activate signaling pathways such as PI3K-AKT, NF-κB, NADH/NADPH oxidase, and PLA2, leading to ROS generation, DNA damage, and inflammatory responses. LCA: activates Erk1/2, which suppresses STAT3 phosphorylation, inducing IL-8 and miRNA-21 expression, thereby contributing to tumor progression.

Recent studies have begun to unravel the carcinogenic role played by microbiota-derived SBAs within the gastrointestinal tract. These microbial metabolites, which are derived from the modification of primary BAs by gut bacteria, have long been recognized for their physiological functions. However, emerging evidence suggests that SBAs exhibiting the potential to contribute to carcinogenesis which is a complex process involving multiple mechanisms (**Table 2**). The mechanism of the carcinogenic effects of SBAs in the development of CRC is shown in **Figure 2B**.

**Table 2:** The carcinogenic effect of SBAs.

Cancer type	Metabolite	Effect	Reference
**Colorectal cancer**	DCA LCA	- Cause miRNA dysfunction and promoting tumor formation, a-1 protein and caspase activation, induce proliferation and DNA damage, upregulated expression of c-fos and cox-2 - ROS production which results in resistance to apoptotic cell death, increased cell proliferation, induces IL-8 expression	(11, 54–56) (53, 57)
**Esophageal cancer**	DCA	- DNA damage, antiapoptotic effect by NFkB activation	(58)
**Gastric cancer**	DCA LCA TLCA	- Stimulation the normal gastric epithelial cells of the rat Upregulated the expression of CDX2 and MUC2 by activating FXR - Promotes the proliferation of normal gastric epithelial cells (GES-1) through activation of the IL-6/JAK1/STAT3 pathway	(59)
**Hepatocellular carcinoma**	DCA LCA	- Activation of FXR and TGR5 signaling	(60, 61)
**Pancreatic cancer**	DCA	- EGFR/Ras/MAPK activation, upregulated expression of cox-2 and AP-1, PKC activation	(62, 63)

### 5.1 Oxidative stress and DNA damage

One carcinogenic effect of microbiota-derived SBAs is their role in promoting DNA damage and mutations. Several studies have demonstrated that these BAs can induce direct DNA damage by activating various plasma membrane enzymes, including phospholipase A2 (PLA2) and NADPH oxidase, leading to the generation of reactive oxygen species (ROS), DNA breaks, and mutations (54, 64–66). Genomic instability is mainly characterized by the emergence of heteroploidy, intrachromosomal instability, and gene point mutations. The elevated risk of CRC is associated with defective DNA repair in response to oxidative damage induced by DCA. Furthermore, an increased concentration of LCA has the potential to cause DNA molecule destruction and hinder the function of DNA repair enzymes (53).

### 5.2 Dysregulation of programmed cell death

SBAs possess the capability to trigger cell death via both nonspecific detergent effects and receptor-mediated interactions. Increased DCA and LCA predominantly induce apoptosis by activating the intrinsic apoptotic pathway. This pathway involves the stimulation of mitochondrial oxidative stress, the generation of ROS, the release of cytochrome C (cytC), and the activation of cytosolic caspases (67). Studies indicated that SBAs may elicit varied cellular responses based on their concentrations. This has led to the possibility that the epithelial cells lining the intestinal tract could develop resistance to apoptosis (45). The persistent exposure of the colonic epithelium to elevated concentrations of SBAs, such as DCA, could facilitate the selective growth of apoptosis-resistant cells, consequently increasing the mutation rate (68–70). The activation of the Nuclear factor kappa B (NF-κB) transcription factor is a crucial survival mechanism that inhibits apoptosis by up-regulating XIAP and Bcl-XL. Numerous studies have highlighted bile acid-induced NF-κB activation in hepatocytes, colorectal cells, and esophageal cells, potentially contributing to the development of cancer (58, 69, 71). Another signaling pathway associated with activated NF-κB is the production of pro-inflammatory cytokines such as TNF, IL-1β and IL-6. The IL-1β subsequently signals either in an autocrine or paracrine manner to activate the phosphatidylinositol 3-kinase (PI3K)- mouse double minute 2 homolog (MDM2) pathway, causing suppression of p53 function. Reduced p53 activity leads to a decrease in apoptosis, alongside the increased survival of damaged DNA cells, and could possibly facilitate the growth of CRC (72, 73). Moreover, IL-6 not only stimulates the Janus kinase (JAK)-signal transducer and activator of transcription 3 (STAT3) pathway, but also promotes the steps leading up to the development of hepatocellular carcinoma. This particular pathway decreases apoptosis and supports the progression of cancer cells (74).

### 5.2 Bile acid receptor-mediated signaling pathways

SBAs can act as ligands for nuclear receptors, such as FXR, pregnane X receptor (PXR), membrane TGR5 receptor, constitutive androstane receptor (CAR), and vitamin D receptor (VDR) (9, 75). With the activation of each receptor resulting in the induction of signaling pathways with various physiological functions, such as proliferation, mitosis, and apoptosis (76). Among the various receptors, there has been extensive research on the activation of FXR and TGR5 by BAs. The FXR-mediated signaling pathways are involved in BA metabolism (9, 77) and the regulation of inflammatory responses (78). Conducted studies indicate the role of FXR in both hepatic (79) and intestinal cancer (80) preventing BA-induced cytotoxicity by sustaining BA concentrations in the physiological range (78). The absence of FXR might be associated with a tumorigenic phenotype; in an animal study, the development of hepatocellular carcinoma had been observed in FXR knockout mice (79). Another receptor is TGR5, which is involved in the regulation of glucose metabolism and energy homeostasis which is represented in the gastrointestinal tract (81). Yang et al. indicated the role of TGR5 activation in the mechanism of BA-induced human hepatocyte apoptosis. In a hepatocyte cell line, TGR5 stimulation was found to promote c-Jun N-terminal kinases (JNK) activation and reduce the formation of the JNK-caspase-8 complex. This, in turn, facilitates caspase-8 recruitment to the death-inducing signaling complex (DISC), triggering apoptosis signaling. These findings suggest that TGR5 may contribute to the genesis and progression of liver diseases by inducing apoptosis in hepatocytes and, consequently, play a role in liver carcinogenesis (82).

The EGFR signaling axis is also crucial for regulating colonic epithelial cell proliferation, apoptosis, and survival, contributing significantly to cellular homeostasis. Two main EGFR pathways, the mitogen-activated protease pathway (Ras/Raf/MEK/ERK/MAPK) and PI3K pathway, are recognized (83). DCA activates the EGFR signaling pathway in colonic epithelial cells, with two proposed mechanisms: stimulation of epidermal growth factor production and release, enhancing receptor activity, and interference with cell membrane structure, activating the receptor. Studies show that DCA induces tyrosine phosphorylation, activating the EGFR pathway in a ligand-dependent manner. This activation involves the Ras/Raf/MEK/ERK/MAPK pathway, leading to AP-1 activation, which mediates cell proliferation and differentiation. Additionally, DCA activates the PI3K/Akt/i-b/NF-B pathway, influencing downstream targets such as the caspase family and NF-B transcription factor, thus regulating cell proliferation and apoptosis (84).

### 5.3 Dysbiosis of gut microbiota

Another cancer-inducing mechanism of BAs is their role in dysbiosis, referring to a modification in the composition of gut microbiota characterized by a decrease in beneficial bacteria and an overgrowth of opportunistic pathogens (85). Experiments have indicated that the elevation of SBAs, particularly DCA, might change the composition of gut microbiota (86, 87) due to their potential antibiotic activity (88). Moreover, DCA has been shown to promote the growth of opportunistic pathogens within the gut microbiota. These include bacteria such as *Shigella*, *Desulforvibrio*, *Ruminococcus*, and *Dorea*. The overgrowth of these opportunistic pathogens has been implicated in the development of CRC (9). A review article has suggested that certain bacteria, which become prevalent after dysbiosis, may have carcinogenic properties. An example is *Streptococcus bovis*, which can thrive in the presence of elevated colonic SBAs. In approximately half of the patients diagnosed with *S. bovis* infections, an incidence of adenoma or CRC has been observed (89). Another outcome of dysbiosis is the production of DNA-damaging agents by certain bacteria. For instance, *Enterococcus faecalis* produces superoxide, leading to the generation of hydrogen peroxide, which can induce DNA damage in colonic epithelial cells (27). BAs, by activating the FXR, can also induce the expression and secretion of inducible nitric oxide synthase and interleukin-18. This action inhibits the proliferation of intestinal bacteria. Studies have revealed a notable rise in Firmicutes and a reduction in Bacteroidetes in the intestinal tract of mice with dysfunctional FXR genes, attributable to heightened bile acid secretion in the absence of a functional FXR receptor. This implies that BAs impact the composition of intestinal flora via the FXR signaling pathway (53).

## 6 Experimental evidence supporting the carcinogenic activities of secondary bile acids

### 6.1 Animal studies implicating microbiota-derived secondary bile acids in cancer development

The carcinogenic effect of SBAs, DCA, was first suggested by cook et al. in, 1939 (90). Since then, animal studies have provided compelling evidence that microbiota-derived SBAs, principally DCA, play a significant role in the development and progression of gastrointestinal system cancers (46). In a study conducted by Prasad et al. the prolonged exposure (10 month) of mice models to a DCA diet, led to the development of colonic tumors compared to mice fed a standard diet without DCA. Their findings align with clinical observations of CRC, suggesting a direct link between increased levels of DCA and carcinogenesis (91). In Hayashi et al. experiment, fecal samples of rat models with and without colon cancer were analyzed. In the colon cancer rats, the percentage of DCA was higher than the control rats, supporting the role of DCA as a promoter in colon carcinogenesis (92). In a study by Cao et al., two groups of *Apc^min/+^* mice were provided with either sterile water or 0.2% DCA, and another group of *Apc^min/+^* (Multiple intestinal neoplasia) mice was administrated with a cocktail of antibiotics to deplete gut microbiota. In *Apc^min/+^* mice, DCA induced tumor growth, and in mice depleted of gut microbiota by antibiotic supplementation, inflammation was decreased, implicating the role of DCA and gut dysbiosis in intestinal carcinogenesis (52). Moreover, animal studies have concluded that high-fat diets for rats lead to an increased level of fecal bile acid, resulting in higher tumorigenesis (93, 94). Although animal studies have provided valuable evidence of the role of SBAs in cancer development, further research is required to fully elucidate the underlying mechanisms.

### 6.2 Epidemiological studies linking increased secondary bile acid levels to cancer risk

Epidemiological studies are crucial to prove the findings observed in animal models and to identify potential therapeutic targets for cancer prevention. Several factors are involved in CRC development, including environmental factors such as diet (high amount of fat and low amount of fiber) (93, 95–99) and genetic predisposition (100, 101). The probable carcinogenic role of dietary fat is the synthesis of BAs in the liver, which enhances the absorption of lipids in the small intestine and is subjected to enterohepatic circulation (102). In the colon, gut microbiota biologically transforms entered BAs, resulting in the formation of SBAs with tumor-promoting activity (102). In Ou et al.’s study, the influence of diet on CRC risk in African Americans with a high risk and in rural native Africans with a low risk of colon cancer was examined. The data provide evidence that SBAs production was higher in African Americans than in Africans, which might be attributed to differences in diet, with African Americans consuming more dietary meat and fat and less food containing fiber (50). Ocvirk et al. assessed the dietary intake of Alaska Natives (AN) with the highest recorded incidence of CRC and Rural Africans (RA) with the lowest CRC risk in healthy middle-aged volunteers. The results support the hypothesis in which the low-fiber, high-fat diet of AN people and exposure to carcinogens derived from diet or environment are associated with a tumor-promoting activity reflected by the high rates of adenomatous polyps in AN people (103). Also, it is stated that the levels of Fecal BA in fecal samples of American and European patients with CRC are significantly higher because of the diet with a high amount of fat and protein than that in Asian and African populations whose diet is less fat and protein and contains more fiber (53, 104). The carcinogenic role of elevated SBAs in animal studies was mentioned earlier. Moreover, several human studies have demonstrated similar results among patients with colon cancer (105–110). In a case-control study conducted by Loftfield et al., the serum of cases collected more than 30 years before CRC diagnosis, and the concentrations of 15 BAs were measured. The results showed that among women, SBAs (DCA, GDCA, GLCA, TDCA) were strongly related to an increased risk of CRC. Although no statistically significant associations were detected for BA amounts among men (111). In another case-control study by Imray et al., the fecal bile acid profiles of patients with CRC, polyps, and controls were compared. The results indicated that patients with polyps and CRC had higher total SBAs compared to control subjects (105).

## 7 Specific types of cancer associated with microbiota-derived secondary bile acids

The potential carcinogenic role of SBAs, such as DCA and LCA, has been studied primarily in the context of gastrointestinal cancers, particularly CRC (112). Although, Other studies have implicated the role of BAs in the development of other gastrointestinal tract malignancies, including Barrett’s metaplasia of the esophagus, pancreas, small intestine, gastric and stomach (27, 76) (**Table 1**).

### 7.1 Colorectal cancer

CRC is considered as one the most prevalent and deadly malignancies worldwide (113), urging a detailed investigation about its multifactorial origins. There have been several epidemiological studies which have indicated the association between increased levels of SBAs in colon cancer (103, 105, 106, 111). The presence of elevated levels of DCA, in particular, has been associated with an increased risk of colorectal adenomas and carcinomas through DNA damage, inflammation, and promotion of cell proliferation (76) (**Figure 2B**).

### 7.2 Pancreatic cancer

The association of BAs in pancreatic cancer development have been demonstrated by several studies (114, 115). Previous studies suggested the over expression of cyclooxy-genase-2 (COX-2) in patients with pancreatic adenocarcinoma (116, 117) and in other experiment by Tucker et al. they observed the induction of COX-2 by BAs such as DCA in human pancreatic cancer cell lines (118). These findings indicate a probable role of BAs in the pathogenesis of pancreatic cancer.

### 7.3 Gastric cancer

The role of SBAs in gastric cancer is an area of ongoing research, and the relationship is complex. While some studies suggest potential links between bile acids and gastric cancer, the mechanisms involved are not fully understood. In a study by Kuwahara et al., BA reflux was suggested to be a risk factor for gastric cancer (119). In an animal study, the modified BA metabolism led to Iron deficiency which promote *H. pylori–i*nduced inflammation and gastric carcinogenesis in mice (120).

### 7.4 Small intestine cancer

The role of SBAs in small intestine cancer is an area that has received relatively less attention compared to their roles in colorectal or pancreatic cancer. Small intestine cancer is a rare malignancy, and the specific mechanisms underlying its development are not as well-understood. However, in an epidemiological study, the majority of small intestinal adenocarcinomas are detected in the duodenum, where BA secretions enter the small intestine. This finding indicated that BAs may have the carcinogenic role in small intestine. Prolonged exposure to high concentrations of BAs may contribute to inflammation or other changes in the small intestine, potentially influencing cancer development (121).

### 7.5 Barrett’s esophagus adenocarcinoma

Barrett’s esophagus is a condition in which the normal squamous epithelial lining of the lower esophagus is replaced by metaplastic columnar epithelium, primarily due to chronic gastroesophageal reflux disease (GERD). Barrett’s esophagus is a known risk factor for the development of esophageal adenocarcinoma. SBAs can be involved in Barrett’s esophagus adenocarcinoma development by induction of oxidative DNA damage and oxidative stress, apoptosis and mutation (58). Clinical studies have suggested the role of esophageal BA exposure by indicating their presence in the esophagi of patients with Barrett’s esophagus adenocarcinoma (122, 123).

### 7.6 Hepatocellular carcinoma

In recent years, the incidence of hepatocellular carcinoma (HCC) is rapidly elevating. According to clinical studies, the alteration of BA profiles has been observed in patients with HCC and *in vitro* human cellular models. In addition, animal studies indicated that BA may affect the hepatocytes. In mice lacking FXR, there is a notable accumulation of BAs, leading to a high incidence of spontaneous HCC. In human *in vitro* models, data on BAs in HCC pathogenesis are intricate but suggest a potential role. Physiological doses of obeticholic acid (OCA) and CDCA in Huh-7 and Hep3B cell models promote epithelial-mesenchymal transition (EMT) through TGF-Beta expression. EMT involves changes in cell shape, loss of polarity, and increased migratory potential, potentially contributing to hepatocyte malignant transformation. Collectively, these findings suggest that BAs may influence cellular phenotype regulation, predisposing to HCC development, but the mechanisms are complex and not fully understood (124).

## 8 Anticancer activities of microbiota-derived secondary bile acids

**Figure 3:**
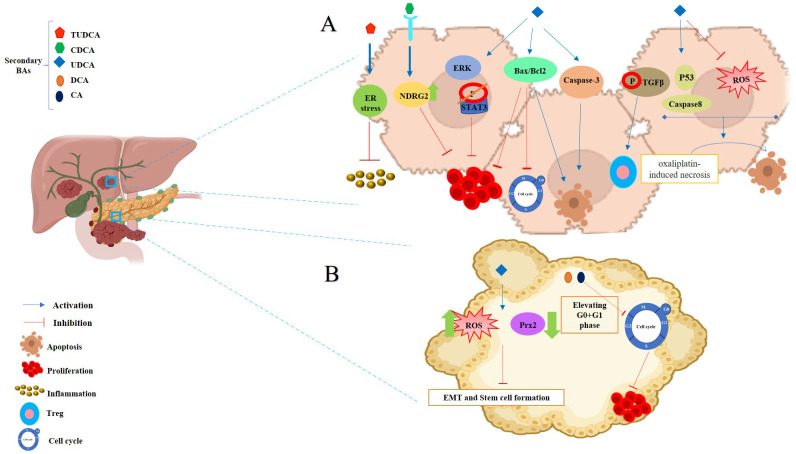
Anticancer effects of SBAs in Hepatocellular Carcinoma (HCC) and Pancreatic Cancer. **(A)** Effects in HCC: TUDCA: mitigates apoptosis induced by ER stress. CDCA: significantly increases NDRG2 expression, inhibiting hepatoma cell proliferation. Phosphorylates TGF-β via the TGR5-cAMP-PKA axis, enhancing T regulatory production. Inhibits cell growth in a dose- and time-dependent manner by increasing the Bax/Bcl-2 ratio and upregulating caspase-3. Disrupts the cell cycle, modulates Bax/Bcl-2 gene expression, and induces apoptosis. Transforms oxaliplatin-induced necrosis into apoptosis by inhibiting ROS generation and activating the p53-caspase 8 pathway. **(B)** Effects in Pancreatic Cancer: UDCA: reduces intracellular ROS, Prx2 levels, epithelial-mesenchymal transition (EMT), and stem cell formation. DCA and CA: cause cell cycle arrest at the G0/G1 phase, effectively inhibiting pancreatic cancer cell proliferation.

**Figure 4:**
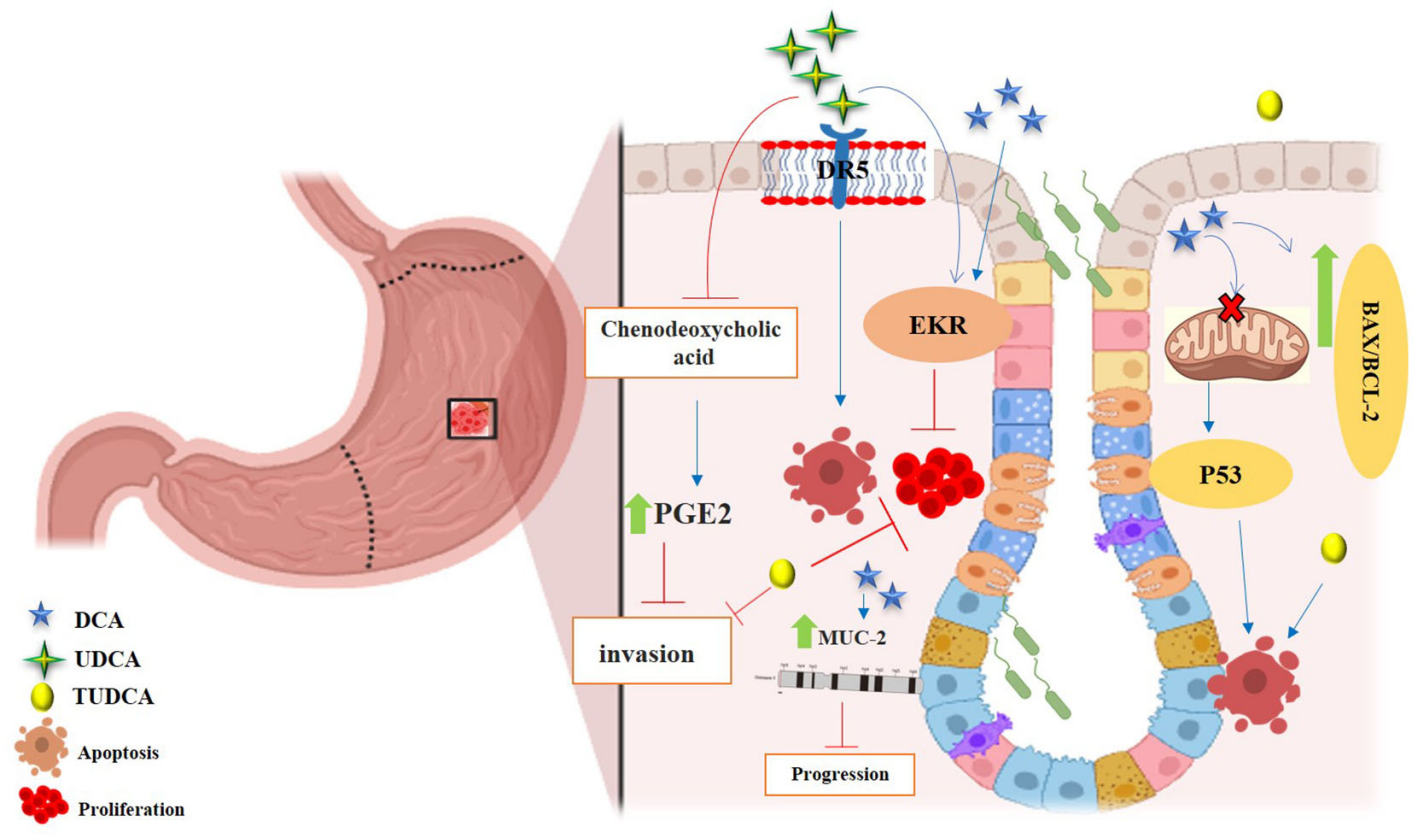
Anticancer effects of SBAs in gastric cancer (GC). TCDCA: suppresses gastric cancer proliferation and invasion while inducing apoptosis. DCA: triggers apoptosis via the intrinsic mitochondrial-dependent, p53-mediated cell death pathway, characterized by an increased Bax/Bcl-2 ratio and disruption of mitochondrial membrane potential. Induces MUC2 expression, which inhibits tumor progression. UDCA: promotes apoptosis and autophagy, overcoming drug resistance. Both UDCA and DCA exhibit suppressive effects on gastric cancer cells by activating ERK signaling pathways. Inhibits invasion by suppressing chenodeoxycholic acid-induced PGE2 production and promotes apoptosis by activating death receptor 5 (DR5) within lipid rafts.

This section will delve into the discussion of the initiation of cancer cell apoptosis and the cytoprotective properties exhibited by SBAs derived from the microbiota. These effects are mediated through multiple signaling pathways, encompassing the modulation of inflammatory responses, interactions with nuclear receptors, initiation of a mitochondrial-associated pathway triggered by ROS culminating in apoptosis, and the regulation of both pro-apoptotic and anti-apoptotic pathways. The mechanism of anticancer action of SBAs in some cancers is shown in **Figures 2A**, **3** and **4**.

### 8.1 LCA derivatives

Persistent inflammation is a recognized contributor to the initiation and progression of cancer. The involvement of Th17 cells and regulatory T cells (Tregs) merits consideration as significant immune factors implicated in the inflammatory pathways associated with cancer (125). Th17 effector cells and Tregs cells located in the lamina propria contribute to immune system homeostasis by producing of a variety of pro-inflammatory mediators and suppressing excessive or inappropriate immune responses, respectively (126). Microbiota-generated BAs may confer protective effects against cancer by regulating inflammatory processes. Two different forms of LCA, namely 3-oxo LCA and isoallo LCA, function as regulators of T cells. 3-oxo LCA inhibits the differentiation of Th17 cells by directly interacting with its crucial transcription factor RORγt (retinoid-related orphan receptor γt), while isoallo LCA boosts Treg differentiation by inducing the generation of mitochondrial reactive oxygen species (mito ROS), resulting in an increase in the expression of Forkhead box P3 (Foxp3) expression (127). Foxp3 acts as an important transcription factor associated with Treg development and differentiation (128). According to a recent study, disrupting the genetic pathways of BAs in gut bacteria decreases the levels of the retinoic acid receptor-related orphan receptor γ (RORγ+) Tregs. Conversely, replenishment of the BAs pool enhances Treg levels and alleviates inflammatory colitis through the activation of BAs nuclear receptors especially the VDR receptor (129).

### 8.2 DCA derivatives

The SBA 3β-hydroxy DCA (iso DCA) can elevate the induction of Foxp3 by influencing dendritic cells (DCs) to reduce their immune-stimulating properties. Eliminating the nuclear receptor FXR in DCs increases the production of Treg cells and imposes a transcriptional profile resembling that induced by iso DCA (130). In addition to T cells, other pathways related to inflammation may also be affected by BAs. Although, previous studies have demonstrated that primary BAs such as 6-ethyl chenodeoxycholic acid and 6a-ethyl-23(S)-methyl cholic acid can inhibit the production of pro-inflammatory cytokines and subsequently suppress NF-kB-dependent signaling pathways (131, 132). The results of the recent study showed that a subset of SBAs, comprising DCA, UDCA, LCA, and TUDCA, can inhibit the activation of caspase-1 and the NOD-, LRR- and pyrin domain-containing protein 3 (NLRP3) inflammasome. The study indicated that BAs can block NLRP3 inflammasome-dependent inflammation, comprising type-2 diabetes-related inflammation, lipopolysaccharide (LPS)-induced systemic inflammation, and alum-induced peritoneal inflammation (133).

### 8.3 TUDCA derivatives

The investigational study conducted by Vandewynckel et al. showed TUDCA decreased inflammation in HCC cells by increasing a key role in regulator of the immune response, the NF-κB inhibitor IκBα as well as suppressing the carcinogen-induced pro-apoptotic unfolded protein response (UPR). TUDCA also decreased eukaryotic initiation factor 2α (eIf2α) phosphorylation, and caspase-12 processing (134). In addition, the chaperoning activity of TUDCA has been reported to be associated with the reduction of endoplasmic reticulum (ER) stress, the stabilization of the UPR and subsequently pro-inflammatory cytokines IL-1β, IL-6 and TNF-α have reported. Upon ER stress, UPR induces the repression of eIf2α phosphorylation and results in a pro-apoptotic state, leading to activate of procaspase-12 (135). Huang et al. reported TUDCA reduced ER stress in adrenocortical carcinoma SW-13 and NCI-H295R cells and induced autophagy through increasing the autophagy factor microtubule-associated protein light chain 3-II/I and the anti-apoptotic B-cell lymphoma 2 (Bcl-2) factor. TUDCA treatment also decreased the expression of the pro-apoptotic factor Bax, resulting in induction of autophagy and inhibition of apoptosis of ACC SW-13 cells after alleviating ER stress (136).

### 8.4 UDCA derivatives

The anti-cancer activity of UDCA on HCC xenografts in mice through increased expression of pro-apoptotic proteins including Bcl-2 associated X protein (Bax), apoptotic protease activating factor-1 (APAF1), cleaved-caspase-9 and cleaved-caspase-3 proteins have reported by Liu et al. (137). Furthermore, UDCA can attenuate the carcinogenesis and cellular invasion of cancer cells induced by primary BA, chenodeoxycholic acid. This highly hydrophobic BA stimulates the invasion of human gastric cancer cells MKN-74 through activation of protein kinase C (PKC) alpha and accordingly increases cyclooxygenase-2 (CoX-2) expression and prostaglandin E2 (PGE2) synthesis. UDCA can suppress PGE2 production and tumor invasiveness induced by CDCA (138). UDCA can also induce apoptosis in human melanoma cell line M14 and A375 through ROS-triggered mitochondrial-associated pathway resulted in increased expression of pro-apoptotic proteins including cleaved-caspase-3, -9, apoptotic protease activating factor-1, and increased Bax/Bcl-2 ratio in treated cells (139).

## 9 Experimental evidence supporting the anticancer activities of secondary bile acids

The cytoprotective properties of SBAs, including UDCA and TUDCA, which decrease the risk of developing colitis-associated cancer and attenuate colon carcinogenesis, particularly in patients with ulcerative colitis, were reported more than 20 years ago (140).

The experimental techniques of MTT (3-(4,5-dimethyl-2-thiazolyl)-2,5-diphenyl-2-*H*-tetrazolium bromide) assay, DAPI (4’,6-diamidino-2-phenylindole) staining, flow cytometry analysis, RT-PCR assay, and western blot assay revealed that UDCA induces apoptotic effects in human oral squamous carcinoma HSC-3 cells by activating caspases. UDCA at concentrations of 400 μg/mL induced apoptosis significantly through increasing of Bax, caspase-3, -8 and -9, TRAIL (TNF-related apoptosis-inducing ligand), IκB-α and DR4, 5 (death receptor) expression, however, the level of Bcl-2 and NF-κB decreased compared to untreated control cell (141).

The apoptosis induction of UDCA on hepatocarcinoma BEL7402 cells injected to BALB/c nude mice was evaluated through detection of DNA fragmentation and the terminal deoxynucleotidyl transferase-mediated dUTP-biotin nick end labeling (TUNEL) assay. The significant suppression of tumor growth and decreasing of tumor volume induced by different concentrations of UDCA were observed over a 21-d period (137).

The anti-tumor activity of UDCA on colon cancer cell lines (HT29 and HCT116) was examined through western blotting, qRT-PCR, and dichlorofluorescin diacetate (DCF-DA) staining. UDCA treatment led to an increase in the expression of cell cycle inhibitors, such as p27 and p21, and the activation of Erk1/2 through the reduction of intracellular ROS in colon cancer. The quantification of tumorsphere-forming potential seven days following treatment with 0.2 mM UDCA showed a decrease in the formation of colon cancer stem-like cells in HT29 and HCT116 cells (142).

A similar study by Kim et al. investigated the effects of UCDA on pancreatic cancer cell lines HPAC and Capan-1. The findings showed that the administration of UDCA led to a reduction in intracellular ROS levels within the pancreatic cancer cells. Moreover, UDCA was observed to decrease both the phosphorylation of STAT3 and the expression of peroxiredoxin II (Prx2). Additionally, the floating-sphere formation assay demonstrated a decline in EMT, as well as a decrease in both the size and quantity of tumorspheres in the pancreatic cancer cells (143).

In another study, researchers have reported on the beneficial role of UDCA in eliminating cisplatin-resistant SNU601 gastric cancer cells by triggering autophagy and apoptosis pathways. The study found that UDCA effectively reduced the viability of cisplatin-resistant SNU601/R cells, which demonstrated strong resistance not only to cisplatin but also to various other anticancer drugs. Moreover, UDCA stimulated autophagic cell death by enhancing the expression of the microtubule-associated protein light chain 3 (LC3II), which serves as an indicator of autophagy, and by increasing the number of autophagic vacuoles, as visualized through monodansylcadaverine (MDC) and HO staining (144).

Wang et al. assessed the impact of UDCA on LC3B expression in both *in vivo* and *in vitro* settings. The results from the cell counting Kit-8 viability assay indicated that UDCA diminished the viability and migration of primary HCC cell lines, 7721 and HepG2. Furthermore, UDCA impeded tumor growth and enhanced LC3B expression in BALB/c nude mice with, 7721 xenografts. By performing Hematoxylin-eosin staining on tumors in nude mice, it was observed that the percentage of cells undergoing cell death increased as the dosage of UDCA increased (145).

The anti-cancer effects of UDCA on FRO human anaplastic thyroid cancer was evaluated through *in vitro* experiments. The findings from the cell viability assay demonstrated that UDCA, at concentrations of 25, 50, and 100 µM/mL, effectively suppressed the growth of cancer cells in a dose-dependent manner. Additionally, UDCA treatment resulted in increased expression of Bax, caspase-3, and cytochrome c, while inhibiting the expression of Bcl-2, TGF-β, and N-cadherin within these cells. Consequently, the administration of UDCA induced apoptosis and hindered the process of angiogenesis by regulating the Akt/mechanistic target of rapamycin (mTOR) signaling pathway (146).

The effects of DCA on the QBC 939 human cholangiocarcinoma cell line and nude mice with xenograft tumors were assessed. The findings revealed that the administration of DCA led to a notable suppression of tumor growth by upregulating the mRNA expression of FXR (147). Barrasa et al. documented the anti-tumor effects of DCA on BCS-TC2 human colon adenocarcinoma cells. These effects were characterized by cell detachment, disruption of membrane asymmetry, and activation of caspase and Bax proteins (148).

Additionally, DCA showed anti-tumor activity on esophageal adenocarcinoma through the IL-6/STAT3 Pathway. The treatment of human EAC, OE33, and normal esophageal HEEC cell lines with 250 µM DCA exhibited an increase in the expression of reprogramming factors Kruppel-like factor and activation of transcription 3 signaling pathway (149). YANG et al. investigated the impact of DCA on BGC-823 human gastric carcinoma cells and explored the underlying mechanisms. Their findings revealed that DCA effectively suppressed cell growth and triggered apoptosis in BGC-823 cells. This apoptotic response was associated with the disruption of mitochondrial membrane potential. Moreover, the expression levels of p53, cyclin D1, and CDK2 were modified after DCA treatment (150). In a recent study, researchers combined the anti-tumor properties of SBAs with the targeted cell selectivity of nanoparticles. The results demonstrated that gold nanoparticles, when combined with polyethylene glycol and LCA connected by carboxyl groups, effectively hindered the growth of HepG2 and SMMC-7721 liver cancer cells. This inhibition was achieved by inducing mitochondrial dysfunction through the mediation of ROS (151).

## 10 Potential influence of secondary bile acid on efficacy of cancer treatment

Secondary bile acids derived from the gut microbiota play a complex role in modulating the efficacy of cancer therapies such as radiation, chemotherapy, and immunotherapy. These bile acids can influence cancer progression and treatment response through various mechanisms, including inhibition of drug transport, and immune modulation. Understanding these interactions is critical to optimizing cancer treatment strategies (152–154). Tumor cells have developed several strategies to evade the damaging effects of chemotherapeutic drugs. One such strategy is the increased expression of ATP-binding cassette (ABC) transporters, which help transport various types of chemotherapeutic drugs out of the cell and into the surrounding environment. Research by Chewchuk et al. found that bile acids, particularly β-cholanic acid and deoxycholic acid, can significantly inhibit ABCC1-mediated drug transport. This leads to a higher accumulation of doxorubicin in breast and lung tumor cells that overexpress ABCC1 and are resistant to the drug. As a result, the IC50 values for doxorubicin in these resistant cell lines were 3 to 4 times lower than in their original parental cell lines. However, these bile acids had no effect on doxorubicin accumulation in drug-sensitive tumor cells lacking ABCC1 expression. This specificity suggests that bile acids could potentially be used as therapeutic agents to combat drug resistance in certain cancers (152). Secondary bile acids have the ability to destabilize the hypoxia-inducible factor-1α (HIF-1α) subunit of the HIF-1 transcription factor. HIF-1 serves as a key transcriptional regulator involved in multiple cancers, and its overexpression under hypoxic conditions is associated with increased tumor aggressiveness, invasiveness, and resistance to standard therapies in several cancer types (155). Phelan et al. found that CDCA and DCA caused destabilization of the HIF-1α subunit in A-549 lung, MCF-7 breast, and DU-145 prostate cancer cell lines, resulting in significantly lower levels of HIF-1α in both normal and hypoxic conditions. This destabilization was validated by immunofluorescence and ELISA techniques, suggesting a possible pathway by which bile acids affect cancer development. The presence of bile acids impaired cancer cell characteristics associated with metastasis, particularly in DU-145 cells, where cell adhesion, migration and invasion were reduced by about half compared to untreated samples. In addition, the clonogenic potential of DU-145 and MCF-7 cells was significantly reduced. Furthermore, CDCA and DCA exhibited minimal cytotoxicity, suggesting that their effects on cancer progression are likely due to alteration of key cancer-associated behaviors rather than induction of cell death (153). Notably, treatment of airway epithelial cells with CDCA and DCA caused a dose-dependent decrease in HIF-1α protein levels, starting at concentrations of 25 μM for CDCA and 1 μM for DCA. In contrast, CA did not affect HIF-1α protein levels, even at the maximum concentration of 100 μM. CDCA and DCA induced the degradation of HIF-1α protein by the 26S proteasome system using the prolyl hydroxylase domain (PHD) pathway (156).

Bile acids have the ability to modify the immune microenvironment, which is critical for the efficacy of immunotherapy. For example, targeting bile acid receptors through nanodelivery can enhance immune responses against tumors in liver cancer, suggesting a promising strategy to improve immunotherapy outcomes. Ji et al. developed nanoparticles containing obeticholic acid (OCA) and 5β-CA (OCA/5β-CA NPs) to target primary and secondary bile acid receptors in the liver. FXR and G protein-coupled bile acid receptor 1 (GPBAR1) are the key receptors for primary and secondary bile acids, respectively (157). The researchers selected OCA as an FXR agonist and 5β-cholanic acid 3 (5β-CA) as a GPBAR1 antagonist to investigate their effects on liver cancer growth. Since FXR and GPBAR1 are prevalent in the gastrointestinal tract, they developed a polyoxazole-based nanosystem to deliver OCA and 5β-CA specifically to the liver, aiming to reduce the potential side effects of these bile acid modulators. The findings revealed that the tumor suppression rates for treatments with OCA-NPs and 5β-CA-NPs were significantly greater than those of the free drug formulations (85% *vs* 48% and 68% *vs* 43%, respectively). Both OCA-NPs and 5β-CA-NPs treatments led to a substantial increase in NKT and NK cells within the orthotopic tumor, along with a notable rise in CXCR6+ NKT cells, suggesting that NKT cells were recruited to the liver via the CXCR6/CXCL16 axis. Additionally, a significant increase in tumor-infiltrating CD4^+^ and CD8^+^ T cells, as well as macrophages, was noted in the treatment groups receiving OCA-NPs and 5β-CA-NPs. There were also considerable increases in levels of IFN-γ and granzyme B in NKT cells, NK cells, CD4^+^, and CD8^+^ T cells in these treatment groups, indicating that NKT cells, NK cells, and T cells all play a role in the tumor inhibition process (154).

## 11 Therapeutic applications of secondary bile acids

Several studies have highlighted the potential utility of SBAs in mitigating damages caused by anticancer drugs, typically conventional cytotoxic agents. The potential therapeutic intervention of UDCA for CRC was reported by Zhang et al. Results of MTT assays and Western blotting showed the stimulation of cAMP-PKA-RhoA pathway to inhibit Yes associated protein (YAP). In addition, UCDA can inhibit HCT116 cells and SW480 cells survival *in vitro* and CRC tumor growth in AOM/DSS-induced primary CRC mice model (158). The results of a prospective randomized trial on twenty-two patients with liver metastases from colorectal carcinoma who received UCDA, or pentoxifylline or low-dose low molecular weight heparin for 8 weeks during radiotherapy revealed significant reduction of the extent and incidence of focal radiation-induced liver injury. The authors proposed the utilization of UCDA, along with two other agents, as a potential strategy for mitigating radiation-induced liver damage following alternative radiotherapeutic interventions (159). It is documented that UCDA can switch necrosis-to-apoptosis in epG2, SK-Hep1, SNU-423 and Hep3B HCC cells when co-treated with oxaliplatin and other platinum-based chemotherapeutic drugs including cisplatin and carboplatin. The co-treatment of UCDA reduced the oxaliplatin-induced ROS generation significantly and induced apoptosis mediated by p53-caspase 8-caspase 3 pathway (160).

In a prospective randomized parallel study, 39 children diagnosed with acute lymphoblastic leukemia (ALL) were chosen to participate. It is well-known that several chemotherapeutic agents used to treat ALL can have harmful effects on the liver (hepatotoxicity). To mitigate this risk, the researchers decided to administer UDCA alongside chemotherapy to the participants for a duration of 6 months. The results showed a promising trend: when UDCA was given in conjunction with chemotherapy, there was a noticeable decrease in levels of hepatic transaminases. This indicates that the combination therapy potentially led to a safer outcome for children with ALL in terms of liver health (161).

The combination of SBAs with anti-cancer drugs for improving the efficacy or decreasing side effects has been documented. Hamano and colleagues evaluated the effectiveness of oral alkalization drugs including UDCA in improving neutropenia following the administration of irinotecan hydrochloride in patients with cervical or ovarian cancer. The use of a drug combination consisting of UDCA, magnesium oxide, and sodium hydrogen carbonate, which are all oral alkalization drugs, had a significant positive impact. It was found that individuals who used this combination experienced improved neutrophil counts and a decreased intensity of the prescribed dosage, when compared to those who did not use the combination (162).

The synergistic effect of UDCA on the antitumor activity of sorafenib was evaluated in HCC Huh-BAT and HepG2 cells. The co-treatment with both agents exerted anti-tumor activities in hepatocytes by inhibiting cell proliferation. Furthermore, the cell viability assay and Annexin V/propidium iodide apoptosis assay revealed induction of apoptosis through ROS-dependent activation of extracellular signal-regulated kinase (ERK) and dephosphorylation of STAT3 (163). The study investigated the effects of low-dose celecoxib and UDCA separately or in combination with primary bile acids on two types of colon cells: HT-29 colon cancer cells and LT97 colorectal micro-adenoma cells derived from a patient with familial adenomatous polyposis. The results demonstrated that the combination of low-dose UDCA and the COX-2 inhibitor celecoxib exhibited inhibitory effects on the growth of colon cancer cells (164). Lin and colleagues showed that treating gallbladder cancer cells with DCA could have a therapeutic effect by affecting the progression and cell proliferation of the cancer cells. This effect is believed to be mediated through the maturation of microRNAs, which is dependent on N6-methyl adenosine. Overall, the findings indicate that DCA treatment might offer a novel therapeutic approach for these types of cancer cells (165). Mikó et al. reported that women with early-stage breast cancer had lower levels of serum LCA, a reduced ratio of CDCA to lithocholic acid, and a decrease in the abundance of the *baiH* gene (which is responsible for generating LCA) in fecal DNA when compared to control women. *In vitro* and *in vivo* examination on MCF7 and 4T1 cell lines, as well as female BALB/c mice, demonstrated that LCA decreased both cancer cell proliferation and metastatic potential of primary tumors (166). The study conducted by Kim et al. revealed the protective effects of UDCA against chemotherapy-induced intestinal mucositis following treatment with 5 fluorouracil (5-FU) in Sprague Dawley rats. The rats were orally administered UCDA for 6 days, resulting in a decrease in levels of inflammatory cytokines and prevention of intestinal villus damage. Moreover, the administration of UDCA led to a significant reduction in body weight loss and diarrhea score compared to the control group during the experiment. The analysis of inflammatory cytokines revealed a significant decrease in the expression of both TNF-α and IL-6 mRNA, which were induced by 5-FU, in the UDCA group (167).

## 12 Conclusion

The complex relationship between microbiota-derived SBAs and cancer underscores their dual role as potential carcinogens and anticancer agents. The carcinogenic properties of SBAs, particularly DCA and LCA, are primarily mediated through mechanisms such as oxidative stress, DNA damage, and induction of dysbiosis within the gut microbiota. These processes can lead to genomic instability, inflammation and the promotion of tumorigenesis, particularly in colorectal cancer. In contrast, certain SBAs also exhibit anticancer activities by inducing apoptosis and modulating immune responses. For example, UDCA has been shown to activate pro-apoptotic pathways and inhibit inflammation, thereby reducing the risk of cancer development. In addition, SBAs can interact with various nuclear receptors and influence cellular signaling pathways that regulate cell proliferation and survival. The balance between these opposing effects highlights the complexity of SBAs in cancer biology and suggests that dietary interventions aimed at modulating bile acid profiles may offer novel therapeutic strategies for cancer prevention and treatment. Future research should focus on elucidating the underlying molecular mechanisms of these dual activities and exploring the potential of SBAs in clinical applications for cancer therapy.

## References

[1] Song M Chan A.T. Environmental factors, gut microbiota, and colorectal cancer prevention. Clin Gastroenterol Hepatol. (2019) 17:275–89. doi: 10.1016/j.cgh.2018.07.012 PMC6314893 30031175

[2] Pasolli E Asnicar F Manara S Zolfo M Karcher N Armanini F. Extensive unexplored human microbiome diversity revealed by over 150,000 genomes from metagenomes spanning age, geography, and lifestyle. Cell. (2019) 176:649–662. e20. doi: 10.1016/j.cell.2019.01.001 30661755 PMC6349461

[3] Belkaid Y Harrison OJJI. Homeostatic immunity and the microbiota. Immunity. (2017) 46:562–76. doi: 10.1016/j.immuni.2017.04.008 PMC5604871 28423337

[4] Ramírez-Pérez O Cruz-Ramón V Chinchilla-López P Méndez-Sánchez J. The role of the gut microbiota in bile acid metabolism. Ann Hepatol. (2018) 16:21–6. doi: 10.5604/01.3001.0010.5494 29080339

[5] Tsilimigras MC Fodor A Jobin C. Carcinogenesis and therapeutics: the microbiota perspective. Nat Microbiol. (2017) 2:1–10. doi: 10.1038/nmicrobiol.2017.8 PMC6423540 28225000

[6] Wilson M. The human microbiota in health and disease: an ecological and community-based approach (1st ed.). (2018). doi: 10.1201/9781351068369

[7] Winston JA Theriot CM. Diversification of host bile acids by members of the gut microbiota. Gut Microbes. (2020) 11:158–71. doi: 10.1080/19490976.2019.1674124 PMC7053883 31595814

[8] Zeng H Umar S Rust B Lazarova D Bordonaro M. Secondary bile acids and short chain fatty acids in the colon: A focus on colonic microbiome, cell proliferation, inflammation, and cancer. Int J Mol Sci. (2019) 20:1214. doi: 10.3390/ijms20051214 30862015 PMC6429521

[9] Yang R Qian L. Research on gut microbiota-derived secondary bile acids in cancer progression. Integr Cancer Ther. (2022) 21:15347354221114100. doi: 10.1177/15347354221114100 35880833 PMC9421216

[10] Kashyap S Pal S Chandan G Saini V Chakrabarti S Saini NK. Understanding the cross-talk between human microbiota and gastrointestinal cancer for developing potential diagnostic and prognostic biomarkers. Semin Cancer Biol. (2022) 86:643–51. doi: 10.1016/j.semcancer.2021.04.020 33971261

[11] Hylemon PB Zhou H Pandak WM Ren S Gil G Dent P. Bile acids as regulatory molecules. J Lipid Res. (2009) 50:1509–20. doi: 10.1194/jlr.R900007-JLR200 PMC2724047 19346331

[12] Axelson M SjÖvall J. Potential bile acid precursors in plasma—possible indicators of biosynthetic pathways to cholic and chenodeoxycholic acids in man. J Steroid Biochem. (1990) 36:631–40. doi: 10.1016/0022-4731(90)90182-R 2214780

[13] Chiang JY Ferrell JM. Bile acids as metabolic regulators and nutrient sensors. Annu Rev Nutr. (2019) 39:175–200. doi: 10.1146/annurev-nutr-082018-124344 31018107 PMC6996089

[14] Wei J Chen T Liu Y Sun S Yuan Z Zhang Y. Targeted bile acids metabolomics in cholesterol gallbladder polyps and gallstones: From analytical method development towards application to clinical samples. J Pharm Anal. (2023) 13:1080–7. doi: 10.1016/j.jpha.2023.06.003 PMC10568091 37842658

[15] Mertens KL Kalsbeek A Soeters MR Eggink HM. Bile acid signaling pathways from the enterohepatic circulation to the central nervous system. Front Neurosci. (2017) 11:617. doi: 10.3389/fnins.2017.00617 29163019 PMC5681992

[16] Vítek L Haluzík M. The role of bile acids in metabolic regulation. J Endocrinol. (2016) 228:R85–96. doi: 10.1530/JOE-15-0469 26733603

[17] Ridlon JM Kang DJ Hylemon PB. Bile salt biotransformations by human intestinal bacteria. J Lipid Res. (2006) 47:241–59. doi: 10.1194/jlr.R500013-JLR200 16299351

[18] Jones BV Begley M Hill C Gahan CG Marchesi JR. Functional and comparative metagenomic analysis of bile salt hydrolase activity in the human gut microbiome. Proc Natl Acad Sci U.S.A. (2008) 105:13580–5. doi: 10.1073/pnas.0804437105 PMC2533232 18757757

[19] Guzior DV Quinn RA. Review: microbial transformations of human bile acids. Microbiome. (2021) 9:140. doi: 10.1186/s40168-021-01101-1 34127070 PMC8204491

[20] Song Z Cai Y Lao X Wang X Lin X Cui Y. Taxonomic profiling and populational patterns of bacterial bile salt hydrolase (BSH) genes based on worldwide human gut microbiome. Microbiome. (2019) 7:9. doi: 10.1186/s40168-019-0628-3 30674356 PMC6345003

[21] Ridlon JM Harris SC Bhowmik S Kang DJ Hylemon PB. Consequences of bile salt biotransformations by intestinal bacteria. Gut Microbes. (2016) 7:22–39. doi: 10.1080/19490976.2015.1127483 26939849 PMC4856454

[22] Osuna-Prieto FJ Xu H Ortiz-Alvarez L Di X Kohler I Jurado-Fasoli L. The relative abundance of fecal bacterial species belonging to the Firmicutes and Bacteroidetes phyla is related to plasma levels of bile acids in young adults. Metabolomics. (2023) 19:54. doi: 10.1007/s11306-023-02016-8 37278866 PMC10244271

[23] Guo X Okpara ES Hu W Yan C Wang Y Liang Q. Interactive relationships between intestinal flora and bile acids. Int J Mol Sci. (2022) 23:8343. doi: 10.3390/ijms23158343 35955473 PMC9368770

[24] Xiao Y Zhao J Zhang H Zhai Q Chen W. Mining genome traits that determine the different gut colonization potential of Lactobacillus and Bifidobacterium species. Microb Genom. (2021) 7:000581. doi: 10.1099/mgen.0.000581 34100697 PMC8461469

[25] Foley MH O'Flaherty S Allen G Rivera AJ Stewart AK Barrangou R. Lactobacillus bile salt hydrolase substrate specificity governs bacterial fitness and host colonization. Proc Natl Acad Sci U.S.A. (2021) 118:e2017709118. doi: 10.1073/pnas.2017709118 33526676 PMC8017965

[26] Wise JL Cummings BP. The 7-α-dehydroxylation pathway: An integral component of gut bacterial bile acid metabolism and potential therapeutic target. Front Microbiol. (2022) 13:1093420. doi: 10.3389/fmicb.2022.1093420 36699589 PMC9868651

[27] Bernstein H Bernstein C. Bile acids as carcinogens in the colon and at other sites in the gastrointestinal system. Exp Biol Med (Maywood). (2023) 248:79–89. doi: 10.1177/15353702221131858 36408538 PMC9989147

[28] Ridlon JM Wolf PG Gaskins HR. Taurocholic acid metabolism by gut microbes and colon cancer. Gut Microbes. (2016) 7:201–15. doi: 10.1080/19490976.2016.1150414 PMC4939921 27003186

[29] Doden H Sallam LA Devendran S Ly L Doden G Daniel SL. Metabolism of oxo-bile acids and characterization of recombinant 12α-hydroxysteroid dehydrogenases from bile acid 7α-dehydroxylating human gut bacteria. Appl Environ Microbiol. (2018) 84:e00235-18. doi: 10.1128/AEM.00235-18 29549099 PMC5930368

[30] Kakiyama G Pandak WM Gillevet PM Hylemon PB Heuman DM Daita K. Modulation of the fecal bile acid profile by gut microbiota in cirrhosis. J Hepatol. (2013) 58:949–55. doi: 10.1016/j.jhep.2013.01.003 PMC3936319 23333527

[31] Hu H Shao W Liu Q Liu N Wang Q Xu J. Gut microbiota promotes cholesterol gallstone formation by modulating bile acid composition and biliary cholesterol secretion. Nat Commun. (2022) 13:252. doi: 10.1038/s41467-021-27758-8 35017486 PMC8752841

[32] Režen T Rozman D Kovács T Kovács P Sipos A Bai P. The role of bile acids in carcinogenesis. Cell Mol Life Sci. (2022) 79:243. doi: 10.1007/s00018-022-04278-2 35429253 PMC9013344

[33] Hirano S Masuda N. Epimerization of the 7-hydroxy group of bile acids by the combination of two kinds of microorganisms with 7 alpha- and 7 beta-hydroxysteroid dehydrogenase activity, respectively. J Lipid Res. (1981) 22:1060–8. doi: 10.1016/S0022-2275(20)40663-7 6946176

[34] Wang S Martins R Sullivan MC Friedman ES Misic AM El-Fahmawi A. Diet-induced remission in chronic enteropathy is associated with altered microbial community structure and synthesis of secondary bile acids. Microbiome. (2019) 7:126. doi: 10.1186/s40168-019-0740-4 31472697 PMC6717631

[35] Lee JY Arai H Nakamura Y Fukiya S Wada M Yokota A. Contribution of the 7β-hydroxysteroid dehydrogenase from Ruminococcus gnavus N53 to ursodeoxycholic acid formation in the human colon. J Lipid Res. (2013) 54:3062–9. doi: 10.1194/jlr.M039834 PMC3793610 23729502

[36] Ferrandi EE Bertolesi GM Polentini F Negri A Riva S Monti D. In search of sustainable chemical processes: cloning, recombinant expression, and functional characterization of the 7α- and 7β-hydroxysteroid dehydrogenases from Clostridium absonum. Appl Microbiol Biotechnol. (2012) 95:1221–33. doi: 10.1007/s00253-011-3798-x 22198717

[37] Liu L Aigner A Schmid RD. Identification, cloning, heterologous expression, and characterization of a NADPH-dependent 7β-hydroxysteroid dehydrogenase from Collinsella aerofaciens. Appl Microbiol Biotechnol. (2011) 90:127–35. doi: 10.1007/s00253-010-3052-y 21181147

[38] Wegner K Just S Gau L Mueller H Gérard P Lepage P. Rapid analysis of bile acids in different biological matrices using LC-ESI-MS/MS for the investigation of bile acid transformation by mammalian gut bacteria. Anal Bioanal Chem. (2017) 409:1231–45. doi: 10.1007/s00216-016-0048-1 27822648

[39] Lee JW Cowley ES Wolf PG Doden HL Murai T Caicedo KYO. Formation of secondary allo-bile acids by novel enzymes from gut Firmicutes. Gut Microbes. (2022) 14:2132903. doi: 10.1080/19490976.2022.2132903 36343662 PMC9645264

[40] Caliceti C Punzo A Silla A Simoni P Roda G Hrelia S. New insights into bile acids related signaling pathways in the onset of colorectal cancer. Nutrients. (2022) 14:2964. doi: 10.3390/nu14142964 35889921 PMC9317521

[41] Wertheim BC Martínez ME Ashbeck EL Roe DJ Jacobs ET Alberts DS. Physical activity as a determinant of fecal bile acid levels. Cancer Epidemiol Biomarkers Prev. (2009) 18:1591–8. doi: 10.1158/1055-9965.EPI-08-1187 PMC2743306 19383885

[42] Pezzali JG Shoveller AK Ellis J. Examining the effects of diet composition, soluble fiber, and species on total fecal excretion of bile acids: A meta-analysis. Front Vet Sci. (2021) 8:748803. doi: 10.3389/fvets.2021.748803 34692814 PMC8529021

[43] Wan Y Yuan J Li J Li H Zhang J Tang J. Unconjugated and secondary bile acid profiles in response to higher-fat, lower-carbohydrate diet and associated with related gut microbiota: A 6-month randomized controlled-feeding trial. Clin Nutr. (2020) 39:395–404. doi: 10.1016/j.clnu.2019.02.037 30876827

[44] Devkota S Wang Y Musch MW Leone V Fehlner-Peach H Nadimpalli A. Dietary-fat-induced taurocholic acid promotes pathobiont expansion and colitis in Il10-/- mice. Nature. (2012) 487:104–8. doi: 10.1038/nature11225 PMC3393783 22722865

[45] Ajouz H Mukherji D Shamseddine A. Secondary bile acids: an underrecognized cause of colon cancer. World J Surg Oncol. (2014) 12:164. doi: 10.1186/1477-7819-12-164 24884764 PMC4041630

[46] Nagengast FM Grubben MJ van Munster IP. Role of bile acids in colorectal carcinogenesis. Eur J Cancer. (1995) 31a:1067–70. doi: 10.1016/0959-8049(95)00216-6 7576993

[47] Bernstein C Holubec H Bhattacharyya AK Nguyen H Payne CM Zaitlin B. Carcinogenicity of deoxycholate, a secondary bile acid. Arch Toxicol. (2011) 85:863–71. doi: 10.1007/s00204-011-0648-7 PMC3149672 21267546

[48] Chen KH Mukaisho K Sugihara H Araki Y Yamamoto G Hattori T. High animal-fat intake changes the bile-acid composition of bile juice and enhances the development of Barrett's esophagus and esophageal adenocarcinoma in a rat duodenal-contents reflux model. Cancer Sci. (2007) 98:1683–8. doi: 10.1111/j.1349-7006.2007.00605.x PMC11158926 17868414

[49] Xie G Wang X Huang F Zhao A Chen W Yan J. Dysregulated hepatic bile acids collaboratively promote liver carcinogenesis. Int J Cancer. (2016) 139:1764–75. doi: 10.1002/ijc.v139.8 PMC5493524 27273788

[50] Ou J Carbonero F Zoetendal EG DeLany JP Wang M Newton K. Diet, microbiota, and microbial metabolites in colon cancer risk in rural Africans and African Americans. Am J Clin Nutr. (2013) 98:111–20. doi: 10.3945/ajcn.112.056689 PMC3683814 23719549

[51] Schulz MD Atay C Heringer J Romrig FK Schwitalla S Aydin B. High-fat-diet-mediated dysbiosis promotes intestinal carcinogenesis independently of obesity. Nature. (2014) 514:508–12. doi: 10.1038/nature13398 PMC4233209 25174708

[52] Cao H Xu M Dong W Deng B Wang S Zhang Y. Secondary bile acid-induced dysbiosis promotes intestinal carcinogenesis. Int J Cancer. (2017) 140:2545–56. doi: 10.1002/ijc.v140.11 28187526

[53] Liu Y Zhang S Zhou W Hu D Xu H Ji G. Secondary bile acids and tumorigenesis in colorectal cancer. Front Oncol. (2022) 12:813745. doi: 10.3389/fonc.2022.813745 35574393 PMC9097900

[54] Powolny A Xu J Loo G. Deoxycholate induces DNA damage and apoptosis in human colon epithelial cells expressing either mutant or wild-type p53. Int J Biochem Cell Biol. (2001) 33:193–203. doi: 10.1016/S1357-2725(00)00080-7 11240376

[55] Kong Y Bai PS Sun H Nan KJ Chen NZ Qi XG. The deoxycholic acid targets miRNA-dependent CAC1 gene expression in multidrug resistance of human colorectal cancer. Int J Biochem Cell Biol. (2012) 44:2321–32. doi: 10.1016/j.biocel.2012.08.006 22903020

[56] Lee HY Crawley S Hokari R Kwon S Kim YS. Bile acid regulates MUC2 transcription in colon cancer cells via positive EGFR/PKC/Ras/ERK/CREB, PI3K/Akt/IkappaB/NF-kappaB and p38/MSK1/CREB pathways and negative JNK/c-Jun/AP-1 pathway. Int J Oncol. (2010) 36:941–53. doi: 10.3892/ijo_00000573 20198339

[57] Kundu S Kumar S Bajaj A. Cross-talk between bile acids and gastrointestinal tract for progression and development of cancer and its therapeutic implications. IUBMB Life. (2015) 67:514–23. doi: 10.1002/iub.v67.7 26177921

[58] Cronin J Williams L McAdam E Eltahir Z Griffiths P Baxter J. The role of secondary bile acids in neoplastic development in the oesophagus. Biochem Soc Trans. (2010) 38:337–42. doi: 10.1042/BST0380337 20298179

[59] Wang S Kuang J Zhang H Chen W Zheng X Wang J. Bile acid–microbiome interaction promotes gastric carcinogenesis. Advanced Sci. (2022) 9:2200263. doi: 10.1002/advs.202200263 PMC9165488 35285172

[60] Maruyama T Miyamoto Y Nakamura T Tamai Y Okada H Sugiyama E. Identification of membrane-type receptor for bile acids (M-BAR). Biochem Biophys Res Commun. (2002) 298:714–9. doi: 10.1016/S0006-291X(02)02550-0 12419312

[61] Wang H Chen J Hollister K Sowers LC Forman BM. Endogenous bile acids are ligands for the nuclear receptor FXR/BAR. Mol Cell. (1999) 3:543–53. doi: 10.1016/S1097-2765(00)80348-2 10360171

[62] Qiao L Studer E Leach K McKinstry R Gupta S Decker R. Deoxycholic acid (DCA) causes ligand-independent activation of epidermal growth factor receptor (EGFR) and FAS receptor in primary hepatocytes: inhibition of EGFR/mitogen-activated protein kinase-signaling module enhances DCA-induced apoptosis. Mol Biol Cell. (2001) 12:2629–45. doi: 10.1091/mbc.12.9.2629 PMC59700 11553704

[63] Glinghammar B Rafter J. Colonic luminal contents induce cyclooxygenase 2 transcription in human colon carcinoma cells. Gastroenterology. (2001) 120:401–10. doi: 10.1053/gast.2001.21188 11159881

[64] Jackson AL Chen R Loeb LA. Induction of microsatellite instability by oxidative DNA damage. Proc Natl Acad Sci U.S.A. (1998) 95:12468–73. doi: 10.1073/pnas.95.21.12468 PMC22854 9770509

[65] Booth LA Gilmore IT Bilton RF. Secondary bile acid induced DNA damage in HT29 cells: are free radicals involved? Free Radic Res. (1997) 26:135–44. doi: 10.3109/10715769709097792 9257125

[66] Craven PA Pfanstiel J DeRubertis FR. Role of reactive oxygen in bile salt stimulation of colonic epithelial proliferation. J Clin Invest. (1986) 77:850–9. doi: 10.1172/JCI112382 PMC423471 3005368

[67] Perez MJ Briz O. Bile-acid-induced cell injury and protection. World J Gastroenterol. (2009) 15:1677–89. doi: 10.3748/wjg.15.1677 PMC2668773 19360911

[68] Glinghammar B Inoue H Rafter JJ. Deoxycholic acid causes DNA damage in colonic cells with subsequent induction of caspases, COX-2 promoter activity and the transcription factors NF-kB and AP-1. Carcinogenesis. (2002) 23:839–45. doi: 10.1093/carcin/23.5.839 12016158

[69] Crowley-Weber CL Payne CM Gleason-Guzman M Watts GS Futscher B Waltmire CN. Development and molecular characterization of HCT-116 cell lines resistant to the tumor promoter and multiple stress-inducer, deoxycholate. Carcinogenesis. (2002) 23:2063–80. doi: 10.1093/carcin/23.12.2063 12507930

[70] Garewal H Bernstein H Bernstein C Sampliner R Payne C. Reduced bile acid-induced apoptosis in "normal" colorectal mucosa: a potential biological marker for cancer risk. Cancer Res. (1996) 56:1480–3. 8603388

[71] Mühlbauer M Allard B Bosserhoff AK Kiessling S Herfarth H Rogler G. Differential effects of deoxycholic acid and taurodeoxycholic acid on NF-kappa B signal transduction and IL-8 gene expression in colonic epithelial cells. Am J Physiol Gastrointest Liver Physiol. (2004) 286:G1000–8. doi: 10.1152/ajpgi.00338.2003 14726307

[72] Jia W Xie G Jia W. Bile acid–microbiota crosstalk in gastrointestinal inflammation and carcinogenesis. Nat Rev Gastroenterol Hepatol. (2018) 15:111–28. doi: 10.1038/nrgastro.2017.119 PMC5899973 29018272

[73] Nag S Qin J Srivenugopal KS Wang M Zhang R. The MDM2-p53 pathway revisited. J Biomed Res. (2013) 27:254. doi: 10.7555/JBR.27.20130030 23885265 PMC3721034

[74] Jung IH Choi JH-K Chung Y-Y Lim G-L Park Y-N Park SW. Predominant activation of JAK/STAT3 pathway by interleukin-6 is implicated in hepatocarcinogenesis. Neoplasia. (2015) 17:586–97. doi: 10.1016/j.neo.2015.07.005 PMC4547407 26297436

[75] Degirolamo C Modica S Palasciano G Moschetta A. Bile acids and colon cancer: Solving the puzzle with nuclear receptors. Trends Mol Med. (2011) 17:564–72. doi: 10.1016/j.molmed.2011.05.010 21724466

[76] Debruyne PR Bruyneel EA Li X Zimber A Gespach C Mareel MM. The role of bile acids in carcinogenesis. Mutat Res. (2001) 480-481:359–69. doi: 10.1016/S0027-5107(01)00195-6 11506828

[77] Inagaki T Choi M Moschetta A Peng L Cummins CL McDonald JG. Fibroblast growth factor 15 functions as an enterohepatic signal to regulate bile acid homeostasis. Cell Metab. (2005) 2:217–25. doi: 10.1016/j.cmet.2005.09.001 16213224

[78] Inagaki T Moschetta A Lee YK Peng L Zhao G Downes M. Regulation of antibacterial defense in the small intestine by the nuclear bile acid receptor. Proc Natl Acad Sci U.S.A. (2006) 103:3920–5. doi: 10.1073/pnas.0509592103 PMC1450165 16473946

[79] Kim I Morimura K Shah Y Yang Q Ward JM Gonzalez FJ. Spontaneous hepatocarcinogenesis in farnesoid X receptor-null mice. Carcinogenesis. (2007) 28:940–6. doi: 10.1093/carcin/bgl249 PMC1858639 17183066

[80] De Gottardi A Touri F Maurer CA Perez A Maurhofer O Ventre G. The bile acid nuclear receptor FXR and the bile acid binding protein IBABP are differently expressed in colon cancer. Dig Dis Sci. (2004) 49:982–9. doi: 10.1023/B:DDAS.0000034558.78747.98 15309887

[81] Baptissart M Vega A Maqdasy S Caira F Baron S Lobaccaro JM. Bile acids: from digestion to cancers. Biochimie. (2013) 95:504–17. doi: 10.1016/j.biochi.2012.06.022 22766017

[82] Yang JI Yoon JH Myung SJ Gwak GY Kim W Chung GE. Bile acid-induced TGR5-dependent c-Jun-N terminal kinase activation leads to enhanced caspase 8 activation in hepatocytes. Biochem Biophys Res Commun. (2007) 361:156–61. doi: 10.1016/j.bbrc.2007.07.001 17659258

[83] Merchant NB Rogers CM Trivedi B Morrow J Coffey RJ. Ligand-dependent activation of the epidermal growth factor receptor by secondary bile acids in polarizing colon cancer cells. Surgery. (2005) 138:415–21. doi: 10.1016/j.surg.2005.06.030 16213893

[84] Jean-Louis S Akare S Ali MA Mash EA Jr. Meuillet E Martinez JD. Deoxycholic acid induces intracellular signaling through membrane perturbations. J Biol Chem. (2006) 281:14948–60. doi: 10.1074/jbc.M506710200 16547009

[85] Belizário JE Faintuch J. Microbiome and gut dysbiosis. Exp Suppl. (2018) 109:459–76. doi: 10.1007/978-3-319-74932-7_13 30535609

[86] Islam KB Fukiya S Hagio M Fujii N Ishizuka S Ooka T. Bile acid is a host factor that regulates the composition of the cecal microbiota in rats. Gastroenterology. (2011) 141:1773–81. doi: 10.1053/j.gastro.2011.07.046 21839040

[87] Wang S Dong W Liu L Xu M Wang Y Liu T. Interplay between bile acids and the gut microbiota promotes intestinal carcinogenesis. Mol Carcinog. (2019) 58:1155–67. doi: 10.1002/mc.22999 PMC6593857 30828892

[88] Taranto MP Perez-Martinez G Font de Valdez G. Effect of bile acid on the cell membrane functionality of lactic acid bacteria for oral administration. Res Microbiol. (2006) 157:720–5. doi: 10.1016/j.resmic.2006.04.002 16730163

[89] Sun J Kato I. Gut microbiota, inflammation and colorectal cancer. Genes Dis. (2016) 3:130–43. doi: 10.1016/j.gendis.2016.03.004 PMC5221561 28078319

[90] Cook J Kennaway E Kennaway N. Production of tumours in mice by deoxycholic acid. Nature. (1940) 145:627–7. doi: 10.1038/145627a0

[91] Prasad AR Prasad S Nguyen H Facista A Lewis C Zaitlin B. Novel diet-related mouse model of colon cancer parallels human colon cancer. World J Gastrointest Oncol. (2014) 6:225–43. doi: 10.4251/wjgo.v6.i7.225 PMC4092339 25024814

[92] Hayashi E Amuro Y Endo T Yamamoto H Miyamoto M Kishimoto S. Fecal bile acids and neutral sterols in rats with spontaneous colon cancer. Int J Cancer. (1986) 37:629–32. doi: 10.1002/ijc.2910370425 3082773

[93] Reddy BS Narisawa T Weisburger J. Effect of a diet with high levels of protein and fat on colon carcinogenesis in F344 rats treated with 1, 2-dimethylhydrazine. J Natl Cancer Institute. (1976) 57:567–9. doi: 10.1093/jnci/57.3.567 988189

[94] Reddy BS Watanabe K Weisburger JH Wynder EL. Promoting effect of bile acids in colon carcinogenesis in germ-free and conventional F344 rats. Cancer Res. (1977) 37:3238–42. 884672

[95] A.C. Society. Cancer facts & figures. The Society (2008).

[96] O'Keefe SJ. Diet, microorganisms and their metabolites, and colon cancer. Nat Rev Gastroenterol Hepatol. (2016) 13:691–706. doi: 10.1038/nrgastro.2016.165 27848961 PMC6312102

[97] O'Keefe SJ. The association between dietary fibre deficiency and high-income lifestyle-associated diseases: Burkitt's hypothesis revisited. Lancet Gastroenterol Hepatol. (2019) 4:984–96. doi: 10.1016/S2468-1253(19)30257-2 PMC6944853 31696832

[98] Ocvirk S Wilson AS Appolonia CN Thomas TK O'Keefe SJD. Fiber, fat, and colorectal cancer: new insight into modifiable dietary risk factors. Curr Gastroenterol Rep. (2019) 21:62. doi: 10.1007/s11894-019-0725-2 31792624

[99] Weisburger JH Reddy BS Rose DP Cohen LA Kendall ME Wynder EL. Protective mechanisms of dietary fibers in nutritional carcinogenesis. Basic Life Sci. (1993) 61:45–63. doi: 10.1007/978-1-4615-2984-2_4 8304953

[100] Rebuzzi F Ulivi P Tedaldi G. Genetic predisposition to colorectal cancer: how many and which genes to test? Int J Mol Sci. (2023) 24:2137. doi: 10.3390/ijms24032137 36768460 PMC9916931

[101] Valle L Vilar E Tavtigian SV Stoffel EM. Genetic predisposition to colorectal cancer: syndromes, genes, classification of genetic variants and implications for precision medicine. J Pathol. (2019) 247:574–88. doi: 10.1002/path.2019.247.issue-5 PMC6747691 30584801

[102] Ocvirk S O’Keefe SJD. Dietary fat, bile acid metabolism and colorectal cancer. Semin Cancer Biol. (2021) 73:347–55. doi: 10.1016/j.semcancer.2020.10.003 33069873

[103] Ocvirk S Wilson AS Posma JM Li JV Koller KR Day GM. A prospective cohort analysis of gut microbial co-metabolism in Alaska Native and rural African people at high and low risk of colorectal cancer. Am J Clin Nutr. (2020) 111:406–19. doi: 10.1093/ajcn/nqz301 PMC6997097 31851298

[104] Hill MJ. Bile, bacteria and bowel cancer. Gut. (1983) 24:871–5. doi: 10.1136/gut.24.10.871 PMC1420134 6618266

[105] Imray CH Radley S Davis A Barker G Hendrickse CW Donovan IA. Faecal unconjugated bile acids in patients with colorectal cancer or polyps. Gut. (1992) 33:1239–45. doi: 10.1136/gut.33.9.1239 PMC1379494 1427378

[106] Cheah PY. Hypotheses for the etiology of colorectal cancer–an overview. Nutr Cancer. (1990) 14:5–13. doi: 10.1080/01635589009514073 2195469

[107] Hill MJ. Bile flow and colon cancer. Mutat Res. (1990) 238:313–20. doi: 10.1016/0165-1110(90)90023-5 2188127

[108] Hill MJ Taylor AJ Thompson MH Wait R. Fecal steroids and urinary volatile phenols in four Scandinavian populations. Nutr Cancer. (1982) 4:67–73. doi: 10.1080/01635588209513740 7155919

[109] Jensen OM MacLennan R Wahrendorf J. Diet, bowel function, fecal characteristics, and large bowel cancer in Denmark and Finland. Nutr Cancer. (1982) 4:5–19. doi: 10.1080/01635588209513733 7155918

[110] Reddy BS Wynder EL. Large-bowel carcinogenesis: fecal constituents of populations with diverse incidence rates of colon cancer. J Natl Cancer Inst. (1973) 50:1437–42. doi: 10.1093/jnci/50.6.1437 4717561

[111] Loftfield E Falk RT Sampson JN Huang WY Hullings A Murphy G. Prospective associations of circulating bile acids and short-chain fatty acids with incident colorectal cancer. JNCI Cancer Spectr. (2022) 6:pkac027. doi: 10.1093/jncics/pkac027 35583137 PMC9115675

[112] Baijal PK Fitzpatrick DW Bird RP. Comparative effects of secondary bile acids, deoxycholic and lithocholic acids, on aberrant crypt foci growth in the postinitiation phases of colon carcinogenesis. Nutr Cancer. (1998) 31:81–9. doi: 10.1080/01635589809514685 9770718

[113] Dekker E Tanis PJ Vleugels JLA Kasi PM Wallace MB. Colorectal cancer. Lancet. (2019) 394:1467–80. doi: 10.1016/S0140-6736(19)32319-0 31631858

[114] Malhotra P Palanisamy R Caparros-Martin JA Falasca M. Bile acids and microbiota interplay in pancreatic cancer. Cancers. (2023) 15:3573. doi: 10.3390/cancers15143573 37509236 PMC10377396

[115] Feng HY Chen YC. Role of bile acids in carcinogenesis of pancreatic cancer: An old topic with new perspective. World J Gastroenterol. (2016) 22:7463–77. doi: 10.3748/wjg.v22.i33.7463 PMC5011662 27672269

[116] Molina MA Sitja-Arnau M Lemoine MG Frazier ML Sinicrope FA. Increased cyclooxygenase-2 expression in human pancreatic carcinomas and cell lines: growth inhibition by nonsteroidal anti-inflammatory drugs. Cancer Res. (1999) 59:4356–62. 10485483

[117] Tucker ON Dannenberg AJ Yang EK Zhang F Teng L Daly JM. 3rd, Cyclooxygenase-2 expression is up-regulated in human pancreatic cancer. Cancer Res. (1999) 59:987–90. 10070951

[118] Tucker ON Dannenberg AJ Yang EK Fahey TJ. III, Bile acids induce cyclooxygenase-2 expression in human pancreatic cancer cell lines. Carcinogenesis. (2004) 25:419–23. doi: 10.1093/carcin/bgh010 14656949

[119] Kuwahara A Saito T Kobayashi M. Bile acids promote carcinogenesis in the remnant stomach of rats. J Cancer Res Clin Oncol. (1989) 115:423–8. doi: 10.1007/BF00393330 PMC12211570 2808479

[120] Noto JM Piazuelo MB Shah SC Romero-Gallo J Hart JL Di C. Iron deficiency linked to altered bile acid metabolism promotes Helicobacter pylori-induced inflammation-driven gastric carcinogenesis. J Clin Invest. (2022) 132:e147822. doi: 10.1172/JCI147822 35316215 PMC9106351

[121] Ross RK Hartnett NM Bernstein L Henderson BE. Epidemiology of adenocarcinomas of the small intestine: is bile a small bowel carcinogen? Br J Cancer. (1991) 63:143–5. doi: 10.1038/bjc.1991.29 PMC1971637 1989654

[122] Gotley DC Morgan AP Cooper MJ. Bile acid concentrations in the refluxate of patients with reflux oesophagitis. Br J Surg. (1988) 75:587–90. doi: 10.1002/bjs.1800750632 3395829

[123] Kauer WK Peters JH DeMeester TR Feussner H Ireland AP Stein HJ. Composition and concentration of bile acid reflux into the esophagus of patients with gastroesophageal reflux disease. Surgery. (1997) 122:874–81. doi: 10.1016/S0039-6060(97)90327-5 9369886

[124] Colosimo S Tomlinson JW. Bile acids as drivers and biomarkers of hepatocellular carcinoma. World J Hepatol. (2022) 14:1730–8. doi: 10.4254/wjh.v14.i9.1730 PMC9521453 36185719

[125] Crusz SM Balkwill FR. Inflammation and cancer: advances and new agents. Nat Rev Clin Oncol. (2015) 12:584–96. doi: 10.1038/nrclinonc.2015.105 26122183

[126] Ivanov II McKenzie BS Zhou L Tadokoro CE Lepelley A Lafaille JJ. The orphan nuclear receptor RORγt directs the differentiation program of proinflammatory IL-17+ T helper cells. Cell. (2006) 126:1121–33. doi: 10.1016/j.cell.2006.07.035 16990136

[127] Hang S Paik D Yao L Kim E Trinath J Lu J. Bile acid metabolites control TH17 and Treg cell differentiation. Nature. (2019) 576:143–8. doi: 10.1038/s41586-019-1785-z PMC6949019 31776512

[128] Devaud C Darcy PK Kershaw MH. Foxp3 expression in T regulatory cells and other cell lineages. Cancer Immunology Immunotherapy. (2014) 63:869–76. doi: 10.1007/s00262-014-1581-4 PMC11028988 25063364

[129] Song X Sun X Oh SF Wu M Zhang Y Zheng W. Microbial bile acid metabolites modulate gut RORγ+ regulatory T cell homeostasis. Nature. (2020) 577:410–5. doi: 10.1038/s41586-019-1865-0 PMC7274525 31875848

[130] Campbell C McKenney PT Konstantinovsky D Isaeva OI Schizas M Verter J. Bacterial metabolism of bile acids promotes generation of peripheral regulatory T cells. Nature. (2020) 581:475–9. doi: 10.1038/s41586-020-2193-0 PMC7540721 32461639

[131] Vavassori P Mencarelli A Renga B Distrutti E Fiorucci S. The bile acid receptor FXR is a modulator of intestinal innate immunity. J Immunol. (2009) 183:6251–61. doi: 10.4049/jimmunol.0803978 19864602

[132] Pols TW Nomura M Harach T Sasso GL Oosterveer MH Thomas C. TGR5 activation inhibits atherosclerosis by reducing macrophage inflammation and lipid loading. Cell Metab. (2011) 14:747–57. doi: 10.1016/j.cmet.2011.11.006 PMC3627293 22152303

[133] Guo C Xie S Chi Z Zhang J Liu Y Zhang L. Bile acids control inflammation and metabolic disorder through inhibition of NLRP3 inflammasome. Immunity. (2016) 45:802–16. doi: 10.1016/j.immuni.2016.09.008 27692610

[134] Vandewynckel Y-P Laukens D Devisscher L Paridaens A Bogaerts E Verhelst X. Tauroursodeoxycholic acid dampens oncogenic apoptosis induced by endoplasmic reticulum stress during hepatocarcinogen exposure. Oncotarget. (2015) 6:28011. doi: 10.18632/oncotarget.v6i29 26293671 PMC4695041

[135] Kusaczuk M. Tauroursodeoxycholate—bile acid with chaperoning activity: molecular and cellular effects and therapeutic perspectives. Cells. (2019) 8:1471. doi: 10.3390/cells8121471 31757001 PMC6952947

[136] Huang X Wu L Kuang Y Li X Deng X Liang X. Tauroursodeoxycholic acid mediates endoplasmic reticulum stress and autophagy in adrenocortical carcinoma cells. Oncol Lett. (2019) 18:6475–82. doi: 10.3892/ol.2019.11057 PMC6888259 31814847

[137] Liu H Xu H-W Zhang Y-Z Huang Y Han G-Q Liang T-J. Ursodeoxycholic acid induces apoptosis in hepatocellular carcinoma xenografts in mice. World J Gastroenterology: WJG. (2015) 21:10367. doi: 10.3748/wjg.v21.i36.10367 26420963 PMC4579883

[138] Wu Y-C Chiu C-F Hsueh C-T Hsueh C-T. The role of bile acids in cellular invasiveness of gastric cancer. Cancer Cell Int. (2018) 18:1–8. doi: 10.1186/s12935-018-0569-0 29942193 PMC5963058

[139] Yu H Fu QR Huang ZJ Lin JY Chen QX Wang Q. Apoptosis induced by ursodeoxycholic acid in human melanoma cells through the mitochondrial pathway. Oncol Rep. (2019) 41:213–23. doi: 10.3892/or.2018.6828 PMC6278461 30542709

[140] Tung BY Emond MJ Haggitt RC Bronner MP Kimmey MB Kowdley KV. Ursodiol use is associated with lower prevalence of colonic neoplasia in patients with ulcerative colitis and primary sclerosing cholangitis. Ann Internal Med. (2001) 134:89–95. doi: 10.7326/0003-4819-134-2-200101160-00008 11177311

[141] Pang L Zhao X Liu W Deng J Tan X Qiu L. Anticancer effect of ursodeoxycholic acid in human oral squamous carcinoma HSC-3 cells through the caspases. Nutrients. (2015) 7:3200–18. doi: 10.3390/nu7053200 PMC4446747 25951128

[142] Kim E-K Cho JH Kim E Kim YJ. Ursodeoxycholic acid inhibits the proliferation of colon cancer cells by regulating oxidative stress and cancer stem-like cell growth. PloS One. (2017) 12:e0181183. doi: 10.1371/journal.pone.0181183 28708871 PMC5510851

[143] Kim YJ Jeong SH Kim E-K Kim EJ Cho JH. Ursodeoxycholic acid suppresses epithelial-mesenchymal transition and cancer stem cell formation by reducing the levels of peroxiredoxin II and reactive oxygen species in pancreatic cancer cells. Oncol Rep. (2017) 38:3632–8. doi: 10.3892/or.2017.6045 29130098

[144] Lim S-C Han SI. Ursodeoxycholic acid effectively kills drug-resistant gastric cancer cells through induction of autophagic death. Oncol Rep. (2015) 34:1261–8. doi: 10.3892/or.2015.4076 26133914

[145] Wang F Qin C Li Y Qu W Liu H Li B. Ursodeoxycholic acid induces autophagy via LC3B to suppress hepatocellular carcinoma *in vivo* and *in vitro* . Int J Clin Exp Pathol. (2017) 10:11805. 31966544 PMC6966075

[146] Jung HW Hwang JH. Anticancer effects of Ursi Fel extract and its active compound, ursodeoxycholic acid, in FRO anaplastic thyroid cancer cells. Molecules. (2021) 26:5309. doi: 10.3390/molecules26175309 34500742 PMC8434085

[147] Dai J Wang H Shi Y Dong Y Zhang Y Wang J. Impact of bile acids on the growth of human cholangiocarcinoma via FXR. J Hematol Oncol. (2011) 4:1–8. doi: 10.1186/1756-8722-4-41 21988803 PMC3207959

[148] Barrasa JI Olmo N Pérez-Ramos P Santiago-Gómez A Lecona E Turnay J. Deoxycholic and chenodeoxycholic bile acids induce apoptosis via oxidative stress in human colon adenocarcinoma cells. Apoptosis. (2011) 16:1054–67. doi: 10.1007/s10495-011-0633-x 21789651

[149] Chen M Ye A Wei J Wang R Poon K. Deoxycholic acid upregulates the reprogramming factors KFL4 and OCT4 through the IL-6/STAT3 pathway in esophageal adenocarcinoma cells. Technol Cancer Res Treat. (2020) 19:1533033820945302. doi: 10.1177/1533033820945302 32869704 PMC7469721

[150] Yang HB Song W Cheng MD Fan HF Gu X Qiao Y. Deoxycholic acid inhibits the growth of BGC-823 gastric carcinoma cells via a p53−mediated pathway. Mol Med Rep. (2015) 11:2749–54. doi: 10.3892/mmr.2014.3004 25434397

[151] Zhao M-X Cai Z-C Zhu B-J Zhang Z-Q. The apoptosis effect on liver cancer cells of gold nanoparticles modified with lithocholic acid. Nanoscale Res Lett. (2018) 13:1–8. doi: 10.1186/s11671-018-2653-8 30269179 PMC6163124

[152] Chewchuk S Boorman T Edwardson D Parissenti AM. Bile acids increase doxorubicin sensitivity in ABCC1-expressing tumour cells. Sci Rep. (2018) 8:5413. doi: 10.1038/s41598-018-23496-y 29615646 PMC5882947

[153] Phelan JP Reen FJ Dunphy N O'Connor R O'Gara F. Bile acids destabilise HIF-1α and promote anti-tumour phenotypes in cancer cell models. BMC Cancer. (2016) 16:476. doi: 10.1186/s12885-016-2528-2 27416726 PMC4946243

[154] Ji G Si X Dong S Xu Y Li M Yang B. Manipulating liver bile acid signaling by nanodelivery of bile acid receptor modulators for liver cancer immunotherapy. Nano Lett. (2021) 21:6781–91. doi: 10.1021/acs.nanolett.1c01360 34382807

[155] Masoud GN Li W. HIF-1α pathway: role, regulation and intervention for cancer therapy. Acta Pharm Sin B. (2015) 5:378–89. doi: 10.1016/j.apsb.2015.05.007 PMC4629436 26579469

[156] Legendre C Reen FJ Woods DF Mooij MJ Adams C O'Gara F. Bile acids repress hypoxia-inducible factor 1 signaling and modulate the airway immune response. Infect Immun. (2014) 82:3531–41. doi: 10.1128/IAI.00674-13 PMC4187831 24914220

[157] De Marino S Festa C Sepe V Zampella A. Chemistry and pharmacology of GPBAR1 and FXR selective agonists, dual agonists, and antagonists. Handb Exp Pharmacol. (2019) 256:137–65. doi: 10.1007/164_2019_237 31201554

[158] Zhang H Xu H Zhang C Tang Q Bi F. Ursodeoxycholic acid suppresses the Malignant progression of colorectal cancer through TGR5-YAP axis. Cell Death Discovery. (2021) 7:207. doi: 10.1038/s41420-021-00589-8 34365464 PMC8349355

[159] Seidensticker M Seidensticker R Damm R Mohnike K Pech M Sangro B. Prospective randomized trial of enoxaparin, pentoxifylline and ursodeoxycholic acid for prevention of radiation-induced liver toxicity. PloS One. (2014) 9:e112731. doi: 10.1371/journal.pone.0112731 25393877 PMC4231047

[160] Lim SC Choi JE Kang HS Si H. Ursodeoxycholic acid switches oxaliplatin-induced necrosis to apoptosis by inhibiting reactive oxygen species production and activating p53-caspase 8 pathway in HepG2 hepatocellular carcinoma. Int J Cancer. (2010) 126:1582–95. doi: 10.1002/ijc.v126:7 19728331

[161] Mohammed Saif M Farid SF Khaleel SA Sabry NA El-Sayed MH. Hepatoprotective efficacy of ursodeoxycholic acid in pediatrics acute lymphoblastic leukemia. Pediatr Hematol Oncol. (2012) 29:627–32. doi: 10.3109/08880018.2012.713083 22889151

[162] Hamano H Mitsui M Zamami Y Takechi K Nimura T Okada N. Irinotecan-induced neutropenia is reduced by oral alkalization drugs: analysis using retrospective chart reviews and the spontaneous reporting database. Supportive Care Cancer. (2019) 27:849–56. doi: 10.1007/s00520-018-4367-y 30062585

[163] Lee S Cho YY Cho EJ Yu SJ Lee JH Yoon JH. Synergistic effect of ursodeoxycholic acid on the antitumor activity of sorafenib in hepatocellular carcinoma cells via modulation of STAT3 and ERK. Int J Mol Med. (2018) 42:2551–9. doi: 10.3892/ijmm.2018.3807 PMC6192782 30106087

[164] van Heumen BW Roelofs HM Te Morsche RH Marian B Nagengast FM Peters WH. Celecoxib and tauro-ursodeoxycholic acid co-treatment inhibits cell growth in familial adenomatous polyposis derived LT97 colon adenoma cells. Exp Cell Res. (2012) 318:819–27. doi: 10.1016/j.yexcr.2012.02.004 22366264

[165] Lin R Zhan M Yang L Wang H Shen H Huang S. Deoxycholic acid modulates the progression of gallbladder cancer through N6-methyladenosine-dependent microRNA maturation. Oncogene. (2020) 39:4983–5000. doi: 10.1038/s41388-020-1349-6 32514152 PMC7314665

[166] Mikó E Vida A Kovács T Ujlaki G Trencsényi G Márton J. Lithocholic acid, a bacterial metabolite reduces breast cancer cell proliferation and aggressiveness. Biochim Biophys Acta (BBA)-Bioenergetics. (2018) 1859:958–74. doi: 10.1016/j.bbabio.2018.04.002 29655782

[167] Kim SH Chun HJ Choi HS Kim ES Keum B Seo YS. Ursodeoxycholic acid attenuates 5−fluorouracil−induced mucositis in a rat model. Oncol Lett. (2018) 16:2585–90. doi: 10.3892/ol.2018.8893 PMC6036549 30008943

